# MRI-PHYSIOL-NIRS: an integrated platform to study kidney oxygenation

**DOI:** 10.1038/s44172-026-00799-x

**Published:** 2026-10-16

**Authors:** Thomas Gladytz, Kathleen Cantow, Jason M. Millward, Sonia Waiczies, Ehsan Tasbihi, Dirk Grosenick, Erdmann Seeliger, Thoralf Niendorf

**Affiliations:** 1 Berlin Ultrahigh Field Facility (B.U.F.F.), Max Delbrück Center for Molecular Medicine in the Helmholtz Association, Berlin, Germany; 2 Institute of Translational Physiology, Charité—Universitätsmedizin Berlin, Berlin, Germany; 3 Experimental and Clinical Research Center, a joint cooperation between the Charité Medical Faculty and the Max Delbrück Center for Molecular Medicine in the Helmholtz Association, Berlin, Germany; 4 Physikalisch-Technische Bundesanstalt (PTB), Berlin, Germany

**Keywords:** Magnetic resonance imaging, Biomedical engineering, Physiology

## Abstract

The kidney always operates close to hypoxia, a pathological condition of O_2_-shortage, due to its unique relationship between blood flow and oxygen demand. Renal hypoxia is central to kidney diseases, where progressive loss of renal function ultimately can no longer sustain life. Magnetic resonance imaging (MRI) can assess renal oxygenation non-invasively in vivo via the relaxation times T_2_^*^ and T_2_, which reflect deoxygenated hemoglobin, but are influenced by several physiological confounders. We developed MRI-PHYSIOL-NIRS, a platform integrating MRI with ground-truth probes that directly measure tissue oxygenation in rat kidneys (PHYSIOL), and near-infrared spectroscopy (NIRS) to estimate oxygenation-related parameters to dissect the relationship between MRI-based measures and true tissue oxygenation. Using aortic occlusion and hyperoxia interventions within the MRI scanner, we investigated correlations between non-invasive MRI-based oxygenation measures and true tissue oxygenation. These results demonstrate the potential for entirely MRI-based measurement of renal oxygenation in health and disease, with ultimate application in patients.

## Abstract (web summary)

Thomas Gladytz and colleagues develop MRI-PHYSIOL-NIRS to improve MR-based assessment of renal oxygenation by integrating MRI with direct oxygen probes and near-infrared spectroscopy in rat kidneys during aortic occlusion and hyperoxia. MRI measures correlate with tissue oxygenation, supporting systematic investigation of additional scenarios toward quantitative MR oximetry.

## Introduction

As life moved from sea to land, survival meant conserving water and clearing waste from metabolic processes. The kidney took on this role—concentrating urine and maintaining fluid and electrolyte homeostasis. Its secret? Energy-intensive tubular reabsorption of sodium and water, powered by oxygen (O₂) supply. Unlike most organs, where O_2_ supply and blood flow are tightly regulated by local metabolic demand, renal perfusion is governed primarily by the need to maintain the glomerular filtration rate (GFR). This filtration-driven perfusion dictates the renal tubular workload, and consequently O_2_ consumption of the kidney. This inverted relationship—where blood flow determines O_2_ demand, rather than responding to it—contributes to chronically low renal tissue partial pressure of *O*_*2*_ (pO_2_): the kidney is always at the brink of O_2_ deprivation and hypoxia^1–5^.

Renal hypoxia plays a central role in the pathophysiology of major kidney diseases, particularly in acute kidney injury (AKI), a syndrome of diverse causes defined by a rapid decline in GFR^1,6–10^. Even subtle imbalances between O_2_ delivery and demand can trigger a vicious cycle of increasing renal O_2_ deficits, promoting the development of chronic kidney disease (CKD). AKI predisposes patients to develop CKD, and CKD renders patients more susceptible to AKI^11,12^. Severe CKD ultimately progresses to end-stage renal disease, when renal function can no longer sustain life^13–15^. AKI and CKD are global killers hidden in plain sight, posing an under-recognized challenge, with a substantial socio-economic burden on healthcare systems. Given the vital role of tissue oxygenation in kidney pathology, renal oxygenation has strong potential as an early diagnostic biomarker in AKI—where it may enable detection of injury before irreversible functional decline occurs—as well as a prognostic biomarker, particularly in patients at increased risk of ischemic injury, patients with early CKD or early diabetic kidney disease. Current clinical practice, however, falls short of this goal. Standard biomarkers—serum creatinine concentration and estimated GFR—reflect kidney damage only after significant functional loss has already occurred. Importantly, none of the current biomarkers reveal any data on renal oxygenation, and thus a critical opportunity for early detection and intervention is missed^9,16–18^. What is needed is a means to monitor renal oxygenation in vivo, and use that information to characterize the state of the kidney along a spectrum from healthy to compromised and at risk of damage.

**Fig. 1:**
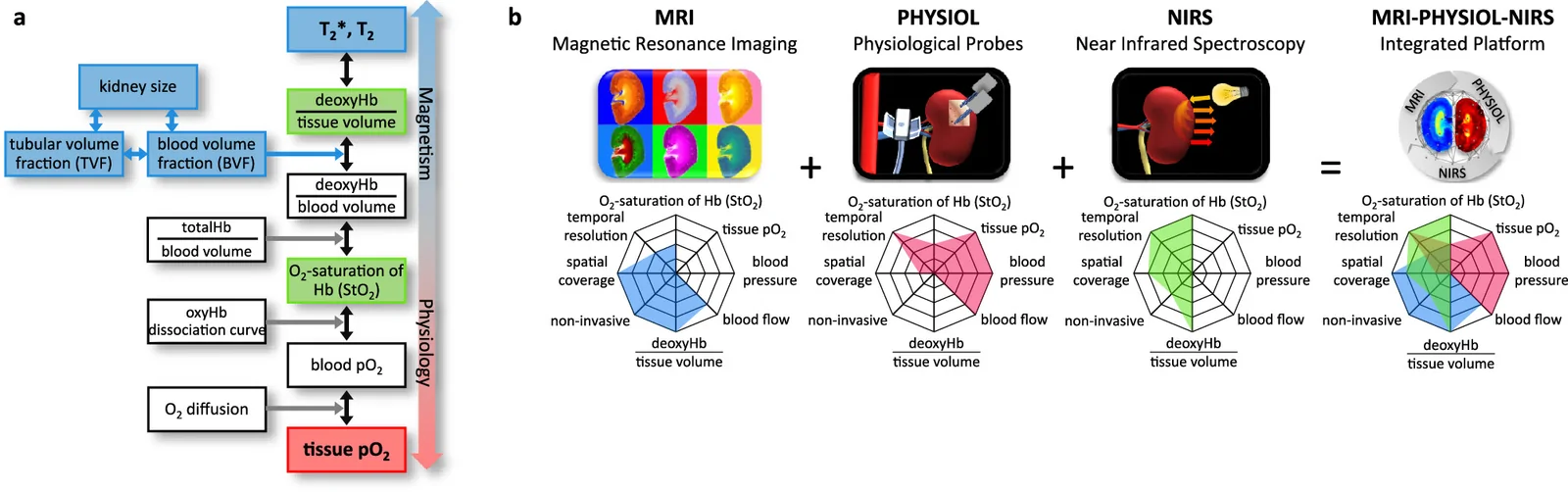
Mechanistic link and basic principle of MRI-PHYSIOL-NIRS. **a** Schematic depiction of the link between apparent renal tissue oxygenation, assessed by MRI parameters T_2_^*^ and T_2_ and true tissue oxygenation, derived from gold-standard measurements of tissue partial pressure of oxygen (pO₂). This link is influenced by deoxygenated hemoglobin (deoxyHb) per tissue and blood volume, O₂ saturation of Hb (StO₂), and blood pO₂. A major confounder is the blood volume fraction (BVF, blood volume per tissue volume), which is affected by changes in tubular volume fraction (TVF) and other factors; both BVF and TVF alterations are often accompanied by changes in kidney size. Additional confounders include totalHb/blood volume (equivalent with hematocrit), the oxyHb dissociation curve, and O₂ diffusion from blood into tissue. Tissue pO₂ (red) can only be measured invasively (PHYSIOL). Confounders measurable by near infrared spectroscopy (NIRS) are shown in green, and those not measurable by MRI or NIRS are shown in white. Parameters in blue are measurable by MRI. **b** Schematic overview of MRI, PHYSIOL, and NIRS modalities, and their integration in the MRI-PHYSIOL-NIRS platform. Spider diagrams illustrate semi-quantitative comparisons of selected physiological parameters (tissue pO₂, StO₂, deoxyHb per tissue volume, blood flow, and arterial blood pressure) and technical parameters (temporal resolution, spatial coverage, and invasiveness). Using five levels, the spider diagrams indicate the approximate extent to which each feature applies. By uniting the distinct yet complementary metrics within the same animal, the MRI-PHYSIOL-NIRS platform provides a unique opportunity to assess the links between MRI metrics and gold-standard oxygenation measures in vivo.

Magnetic resonance imaging (MRI) is a cornerstone of diagnostic imaging, offering non-invasive, high-resolution assessment of anatomy with whole-organ coverage. It can also probe tissue oxygenation in vivo, as deoxygenated hemoglobin (deoxyHb) is paramagnetic and alters MRI signals^5^. Accordingly, transverse relaxation times T_2_ and T_2_^*^ are sensitive to deoxyHb levels and are used as indirect markers of tissue oxygenation^19,20^. However, the relationship of these metrics with true tissue oxygenation (pO_2_) is confounded by several physiological factors including renal blood and tubular volume changes that affect kidney size and signal interpretation (Fig. 1a)^21^. Clear mechanistic links between MRI metrics of apparent oxygenation and true tissue oxygenation remain lacking^5^. Aside from practical constraints including scanner availability and costs, these discrepancies are a major reason why clinical use of MRI for renal oximetry has been limited^5^.

The current *gold standard* for measuring tissue oxygenation in vivo is invasive probes that provide absolute measurements of tissue pO_2_ (Fig. 1a)^3^. Measurements of physiological variables including tissue pO_2_, renal blood flow, and arterial blood pressure (PHYSIOL) (Fig. 1b) have yielded important insights into the role of hypoxia in animal models of kidney disease^22–30^. However, the pO_2_ probes have two major drawbacks. First, they must penetrate into the tissue, and this invasive nature makes them entirely unsuitable for use in humans. Second, the probes measure tissue pO_2_ only at the pinpoint site of insertion (Fig. 1b). This creates sampling bias, since renal tissue pO_2_ displays considerable spatial heterogeneity even within the major compartments (renal cortex and medulla)^3,5^. Overcoming these limitations requires an approach that can measure tissue oxygenation throughout the entire organ, in a non-invasive way that can be readily translated into clinical practice.

Near infrared spectroscopy (NIRS) can bridge the gaps of PHYSIOL and MRI, at a size scale intermediate between the pinpoint measurements of pO_2_ probes, and the whole-organ coverage of MRI (Fig. 1b). Moreover, NIRS allows the estimation of several physiological parameters that confound the relationship between the MRI-based parameters T_2_^*^ and T_2_ and the pO_2_ probe-based true tissue oxygenation (Fig. 1a). These parameters include oxygenated, deoxygenated and total hemoglobin per tissue volume (oxyHb, deoxyHb, totalHb), and O_2_ saturation of hemoglobin (StO_2 NIRS_)^31^.

A meaningful interpretation of the MRI-based markers of oxygenation requires that we understand their relationship with true tissue oxygenation. Unraveling this link requires the integration and simultaneous measurement of parameters obtained from MRI, NIRS, and the invasive physiological probes which provide the ground truth. To accomplish this, we engineered MRI-PHYSIOL-NIRS: a platform that integrates these complementary methods, to decipher the relationship between true and apparent tissue oxygenation (Fig. 1b). This platform allows for physiologically relevant interventions to be applied—while the living animal is inside the MR scanner—to directly compare how changes in MRI metrics, especially T_2_^*^ and T_2_, are related to changes in the quantitative measures of tissue and blood oxygenation from PHYSIOL and NIRS. As proof-of-concept, we demonstrate how an acute pathophysiological intervention (transient occlusion of the suprarenal aorta) and a therapeutic intervention (increase in the inspiratory O_2_ fraction) impacts renal pO_2_, T_2_^*^and T_2_ along with MRI-derived kidney size, tubular volume fraction (TVF), blood volume fraction (BVF) and StO_2 MRI_. Simultaneously capturing information from these distinct and complementary modes using the MRI-PHYSIOL-NIRS platform (Fig. 1b) within the same animal enables an unprecedented opportunity to decode the links between the MRI metrics T_2_^*^, T_2_ and the physiological measures of renal tissue oxygen, while avoiding confounding effects of inter-individual differences that arise when using sequential examinations with stand-alone PHYSIOL, NIRS or MRI.

## Results

### MRI-PHYSIOL-NIRS systems integration and experimental validation

**Fig. 2:**
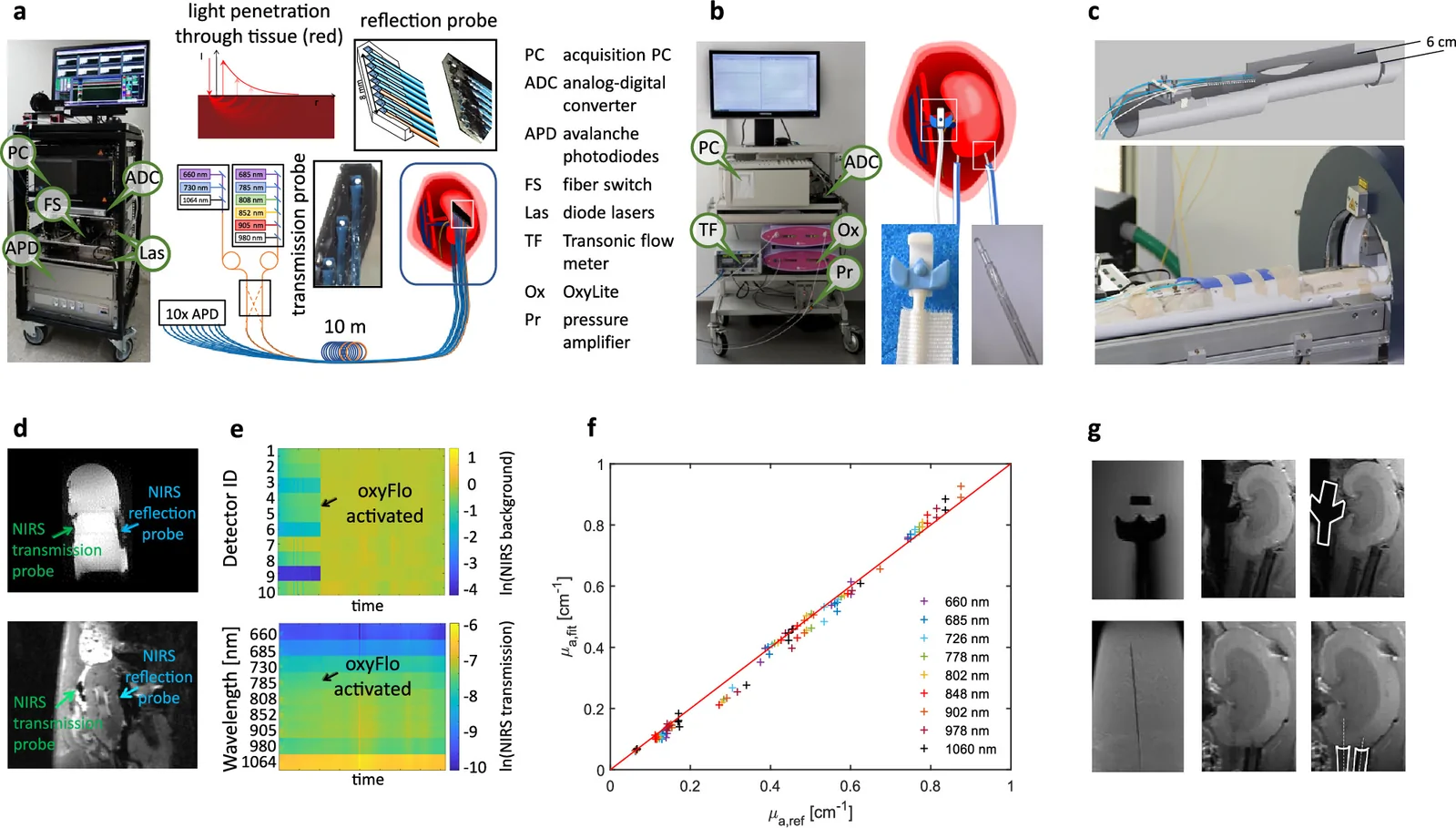
Systems integration and experimental validation. **a** Photograph of the NIRS rack (left) and schematic view of the optical setup of the NIRS system (right). Flexible fibers guide the light from outside the 5 Gauss line of the scanner to the rat kidney and vice versa. Fibers of the transmission and reflection probes are glued into a precisely machined scaffold together with 90° reflective prisms. This allows optical access to the rat kidney within the spatial constraints of the MRI bore and ensures stable alignment. **b** Photographs of the PHYSIOL rack (left), the MR-compatible ceramic perivascular flow probe (middle), and the combined pO_2_/laser flux (oxyLite/oxyFlo) probe (right). **c** Customized animal holder used to safely transport the rat together with the probes into the MR scanner. **d** MR images showing the NIRS probes as compact signal voids in an agarose phantom (upper) and in vivo around the rat kidney (lower); next to the signal voids showing the actual position of the probes, there are only mild susceptibility artifacts confined within 500 µm around the probe, **e** Time traces of the background signal and the NIRS transmission signal after background removal. Light from the laser flux probes (oxyFlo) reaches the NIRS probes, which is compensated, by subtracting the background signal acquired with all NIRS Lasers switched off for 18 ms during every NIRS acquisition cycle. **f** Comparison of the integrated NIRS-derived absorption coefficients of the 11 calibration phantoms with reference values known from gold standard time-resolved NIRS measurements in a leave-one-out cross-validation. **g** Spin-echo MRI images of the perivascular flow probe (upper) and the oxyLite/oxyFlo probe (lower). The probes provided no ^1^H MR signal or artifact, making them visible in the MR images as concise signal voids used for monitoring of the correct position.

NIRS-MRI: To ensure MRI safety, all NIRS electronics were placed outside the 5 Gauss line in a rack containing the data acquisition computer, analog-to-digital converter board, fiber switch, lasers, avalanche photodiodes (APD), and power supply (Fig. 2a). Optical fiber extensions guided light to the kidney and back to the detectors, preventing interference with the electromagnetic fields of the MRI. To avoid displacement of the kidney or strain on the renal vasculature, highly flexible fibers (*D*_core_ = 200 µm) were used. Placing optical fibers perpendicularly on the kidney surface is not possible due to the space constraints of the MRI bore. Therefore, the light was guided into the tissue through 90° reflective prisms which were glued with the fibers onto a scaffold (Fig. 2a). A customized animal holder (Fig. 2c) was designed to fix and stabilize the probes, ensuring safe transport of the animal into the MR scanner. The MR compatibility of the fiber probe head was verified in phantom experiments and in vivo (Fig. 2d). The fiber probe head did not provide any ^1^H MR signal, and thus resulted in a signal void in the MRI images (Fig. 2d), which were used to monitor the probe positioning (Fig. 2e). The thin reflective coating on the prisms (size: 0.5 mm × 0.7 mm) is the only metallic component of the probe, which induced minor MRI susceptibility artifacts in the sub-millimeter range at the interface between the probe head and the kidney (Fig. 2d). To overcome this constraint, coronal imaging slices of the kidney were used for the in vivo experiments.

NIRS-PHYSIOL: Light emitted by the pO_2_- and laser-flux probes of the PHYSIOL setup can reach the NIRS detectors (Fig. 2e) and confound the signal. To overcome this, the background signal induced by the pO_2_/flux probes was quantified at times when the NIRS lasers were off during the switch from one NIRS source fiber to another. The background signal was in the range of 16 mV on the most affected APD, and was subtracted afterwards from all NIRS signals (Fig. 2e). Due to the reduced scattering and absorption during aortic occlusion the background signal in some cases rose to 50 mV, which still left 98% of the dynamic range of the APD available for the actual signal. The influence of the NIRS light on the laser flux signal was minimized by reducing the NIRS laser power to 20 mW and by placing the NIRS probe on the cranial half of the kidney, and the pO_2_/flux probe at the caudal region, maximizing the distance between both probes. We validated the integrated NIRS system by leave-one-out cross-validation on 11 distinct reference phantoms, whose absorption and scattering coefficients are known from gold-standard time-resolved NIRS measurements. For the integrated NIRS-derived absorption coefficients we obtained a mean absolute percentage error of 4% versus the reference (Fig. 2f).

PHYSIOL-MRI: To ensure MRI safety, all PHYSIOL electronics (Fig. 2b) were placed outside the 5 Gauss line in a rack containing the amplifier, flowmeter, and OxyLite/OxyFlo apparatus. Extension lines connected the probes to bridge the distance between the MRI scanner isocenter and the devices. To monitor arterial blood pressure and heart rate, a catheter was inserted into the femoral artery. A long catheter (>1 m) was used to place the pressure transducer outside the magnet bore, where the magnetic fringe field does not affect it. To measure total renal blood flow, the conventional U-shaped brass reflector of the perivascular flow probe was replaced by a reflector made of macor ceramics (Fig. 2b) to ensure MRI compatibility. This L-shaped reflector did not induce signal or artifacts in ^1^H MRI (Fig. 2g). For local tissue oxygenation and local perfusion measurements, we positioned the cortical pO_2_/flux probe differently compared to our previous works. Instead of advancing the probe from the caudal extent centrally through the left kidney to the cortical layer of the cranial extent, it was placed at a depth of approximately 1.5 mm in the caudal extent (Fig. 2g). MRI signal voids induced by the fiber optic probes were minor due to the small probe diameter (0.5 mm). The signal voids appear as straight lines in the caudal extent of the kidney (Fig. 2g), and were used to visualize and determine the correct position of the probes.

**Fig. 3:**
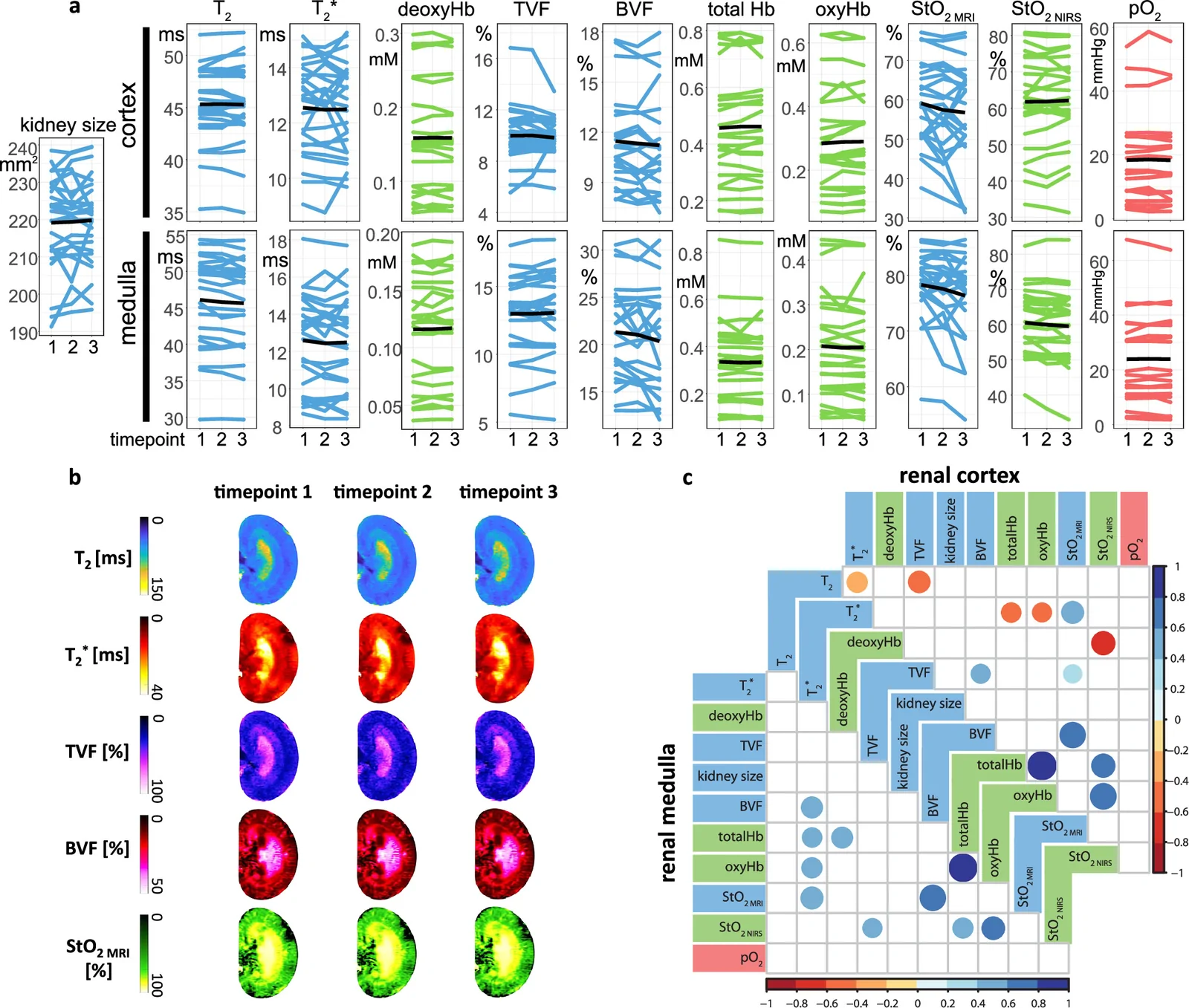
In vivo feasibility study—Baseline. **a** Individual rat (*n* = 25) time courses of the parameters simultaneously acquired by the MRI-PHYSIOL-NIRS platform at three consecutive measurements during the baseline period (7 min), prior to intervention (blue = MRI-based measures, green = NIRS-based measures, red = PHYSIOL-based measure). No statistically significant differences were detected among the three measurements for any of the parameters (all *p*-values > 0.05, repeated measurements ANOVA). **b** Parametric maps from one representative rat, showing consistency of the MR-based results across the three consecutive baseline measurements for T_2_^*^, T_2_, tubular volume fraction (TVF), blood volume fraction (BVF) and O_2_ saturation of hemoglobin (StO_2 MRI_). **c**) Matrices depicting repeated-measures correlation results among all parameters, for medulla and cortex separately. Color and size of the points are scaled to the correlation coefficient; only statistically significant correlations are depicted (repeated-measures correlation with Benjamini-Hochberg correction for multiple comparisons). The list of all correlation coefficients and *p*-values is provided in Supplementary Table 1. All tests are two-sided.

We validated the feasibility of the MR-PHYSIOL-NIRS platform in vivo by examining the intra-individual repeatability at baseline under physiological conditions (*n* = 25), and demonstrated high consistency, reliability and validity (Fig. 3a). The recordings of all parameters throughout the baseline period were stable, showing only small physiological intra-individual fluctuations (Fig. 3a, Table 1). These results are consistent with data we previously obtained in stand-alone settings of the respective modalities: the present MRI data on cortical and medullary T_2_^*^, T_2_, and TVF, StO_2 MRI_ and BVF, and on kidney size quantitatively reflect earlier data and estimates^21,32,33^. The present PHYSIOL data on cortical and medullary pO_2_, arterial blood pressure and renal blood flow are also consistent with earlier results^29^. The present NIRS data on cortical StO_2 NIRS_ reflect earlier data, while data on cortical totalHb are in the same order of magnitude as previous data^31^. These baseline data indicate that the integration of the three modalities in the MRI-PHYSIOL-NIRS platform was successful. The quantitative maps obtained for T_2_^*^, T_2_, TVF, BVF and StO_2 MRI_ demonstrate that the measured parameters are not uniform throughout the kidney, but differ depending on the anatomical compartments including the renal cortex and medulla (Fig. 3b). The correlation analysis among the MRI-PHYSIOL-NIRS parameters reflects the *inter-individual variability of the absolute values*, and yielded few significant correlations (Fig. 3c, Table S1).

**Table 1:** Mean parameters at baseline

	Baseline Mean values and standard errors		Coefficient of variance of three baseline exams	
Heart rate	446 ± 7 min^−1^		0.7 %	
Mean arterial pressure	80.3 ± 2.5 mmHg		2.8 %	
Renal blood flow	6.3 ± 0.6 ml/min		1.8 %	
Respiratory rate	107.0 ± 2.4 min^−1^		2.6 %	
	renal cortex	renal medulla	renal cortex	renal medulla
Transversal relaxation time (T_2_)	45.3 ± 0.7 ms	46.2 ± 1.3 ms	0.8 %	1.0 %
Effective transversal relaxation time (T_2_^*^)	12.5 ± 0.3 ms	12.6 ± 0.5 ms	2.7 %	2.7 %
DeoxyHb per tissue volume	0.16 ± 0.02 mmol/l	0.13 ± 0.02 mmol/l	2.7 %	2.3 %
Tubular volume fraction (TVF)	26.9 ± 1.0 %	34.2 ± 1.5 %	4.6 %	2.7 %
Kidney size (cross section area)	219.6 ± 2.2 mm²		1.1%	
Blood volume fraction (BVF)	11.3 ± 0.6 %	21.3 ± 1.1 %	3.3 %	4.3 %
TotalHb per tissue volume	0.44 ± 0.04 mmol/l	0.33 ± 0.03 mmol/l	2.0 %	2.9 %
OxyHb per tissue volume	0.28 ± 0.03 mmol/l	0.20 ± 0.02 mmol/l	4.3 %	4.4 %
StO_2 MRI_	57.7 ± 2.5 %	78.0 ± 1.4 %	6.4 %	2.6 %
StO_2 NIRS_	61.9 ± 2.5 %	60.0 ± 1.9 %	2.2 %	2.0 %
Tissue pO_2_	19 ± 3.1 mmHg	22 ± 3.4 mmHg	4.3 %	4.4 %

Synopsis of results obtained in vivo at baseline and the repeatability of simultaneous MRI-PHYSIOL-NIRS measurements quantified by the coefficients of variance of three consecutive baseline measurements.

### Acute pathophysiological intervention: transient occlusion of the suprarenal aorta

We investigated the capabilities of the MRI-PHYSIOL-NIRS platform to assess renal oxygenation using an acute pathophysiological and clinically relevant challenge (intrarenal hypoxia: transient occlusion of the suprarenal aorta, *n* = 14), and a therapeutic intervention (hyperoxia: increasing the inspiratory oxygen fraction, *n* = 11). These interventions were chosen because they result in contrasting changes of renal oxygenation *versus* normal physiological conditions. With an expected pO_2_ decrease during aortic occlusion (ΔpO_2_ ∼ −80%) and an expected increase during hyperoxia (ΔpO_2_ > 100%), this experimental design was well suited to showcase the capabilities of MRI-PHYSIOL-NIRS Table 2.

**Table 2:** List of abbreviations

MRI	magnetic resonance imaging
NIRS	near infrared spectroscopy
PHYSIOL	invasive physiological probes
deoxyHb	deoxygenated hemoglobin concentration
oxyHb	oxygenated hemoglobin concentration
totalHb	total hemoglobin concentration
StO_2_	oxygen saturation of the total hemoglobin
TVF	tubular volume fraction
BVF	blood volume fraction
T_2_*	effective transversal relaxation time
T_2_	transversal relaxation time
pO_2_	partial pressure of oxygen (equivalent to oxygen concentration physically dissolved in tissue)
O_2_	oxygen
GFR	glomerular filtration rate
AKI	acute kidney injury
CKD	chronic kidney disease
APD	avalanche photodiode
SEM	standard error of the mean

**Fig. 4:**
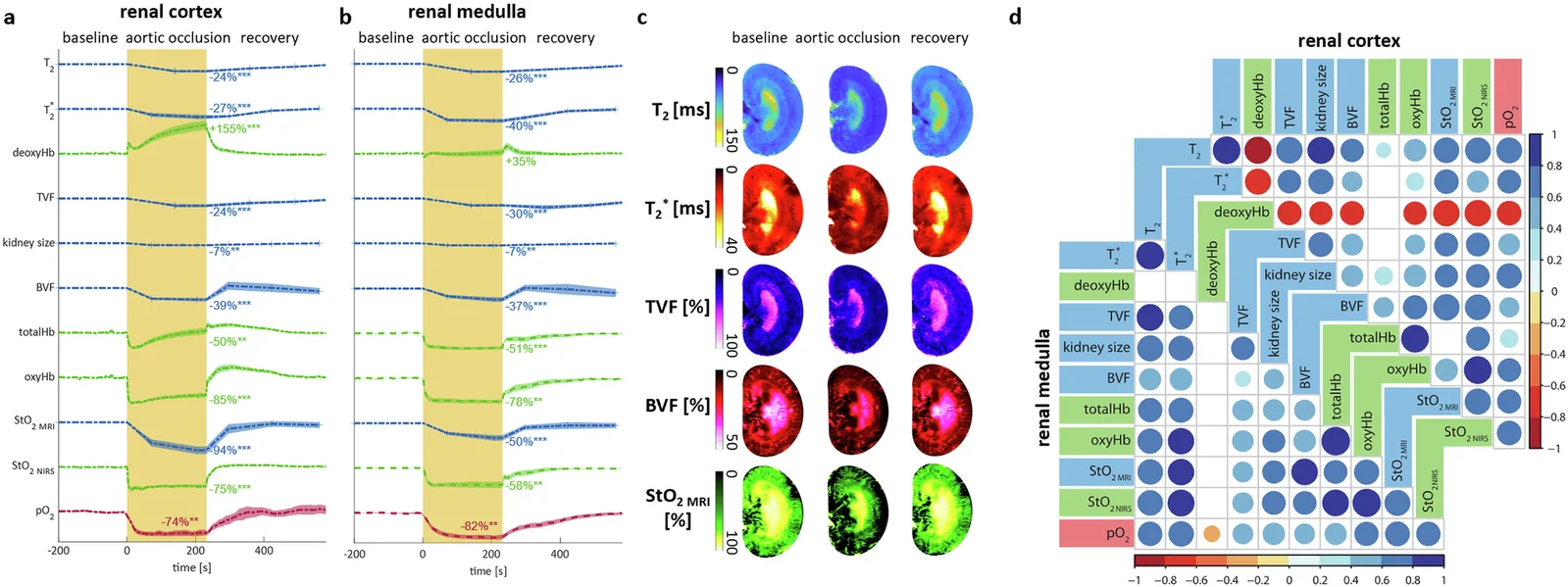
In vivo feasibility study—aortic Occlusion. Time courses of the parameters simultaneously acquired by the MRI-PHYSIOL-NIRS platform during aortic occlusion (yellow shaded area) and recovery for **a** renal cortex and **b** renal medulla (blue = MRI-based measures, green = NIRS-based measures, red = PHYSIOL-based measure). Values are depicted as mean ± SEM (*n* = 14), scaled as percent change relative to baseline for each parameter separately. The numbers in the plots show the maximum percent change relative to the baseline (**p < 0.01, ***p < 0.001, non-parametric repeated-measures Friedman test followed by Dunn’s procedure). **c** Parametric maps from one representative rat, illustrating changes during the aortic occlusion for T_2_^*^, T_2_, TVF, BVF and StO_2 MRI_, and the restoration of these MR measures during the recovery phase. **d** Matrices depicting repeated-measures correlation results among all parameters, for renal medulla and cortex separately. Color and size of the points are scaled to the correlation coefficient; only statistically significant correlations are depicted (repeated-measures correlation with Benjamini-Hochberg correction for multiple comparisons). The list of all correlation coefficients and *p*-values is provided in Supplementary Tables 1 and 2. All tests are two-sided.

Several surgical procedures require cross-clamping of the renal artery or suprarenal aorta, for example during kidney transplantation^34–36^. If maintained for too long, such occlusions risk severe intrarenal hypoxia and acute renal ischemia-reperfusion injury^29,37^. Aortic occlusion resulted in significant changes in all measured parameters, with the exception of medullary deoxyHb (Fig. 4a, b). Upon aortic occlusion, pO_2_, MRI-derived and spatially resolved T_2_^*^, T_2_, StO_2 MRI_, BVF, TVF and kidney size decreased (Fig. 4a–c) along with NIRS-derived StO_2 NIRS_, totalHb, and oxyHb, while cortical NIRS-derived deoxyHb increased (Fig. 4a, b). Upon release of the occlusion, most parameters returned to pre-occlusion levels within 4 min (Fig. 4a, b). These in vivo results show that T_2_^*^, T_2_ changes during aortic occlusion and recovery are primarily driven by tissue oxygenation: the analysis showed significant correlations between pO_2_ and T_2_^*^, T_2_ (Fig. 4d, Table S1). Moreover, significant correlations were observed among all oxygenation-related parameters (pO_2_, T_2_^*^, T_2_, StO_2 MRI_, StO_2 NIRS_ and deoxyHb) for the cortex and—except for deoxyHb—also for the medulla (Fig. 4d, Table S1). This indicates alignment of all variables that constitute links between true tissue oxygenation and the surrogates T_2_^*^, T_2_. However, the correlations of changes of T_2_^*^, T_2_ with those of BVF, TVF, and kidney size indicate that TVF and BVF have a considerable impact on the relationship between true and apparent oxygenation. Thus, physiological interpretation of T_2_^*^, T_2_ as markers for renal tissue oxygenation must account for changes in these variables. While changes in kidney size are easily quantifiable from T_2_^*^ and T_2_ images, they do not *per se* allow differentiation between changes induced by either TVF or BVF. Thus, our data suggest that MRI-based quantification of TVF and/or BVF should routinely be performed together with kidney size for physiological interpretation of renal T_2_^*^, T_2_.

### Therapeutic intervention: hyperoxia due to increase in the inspiratory oxygen fraction

**Fig. 5:**
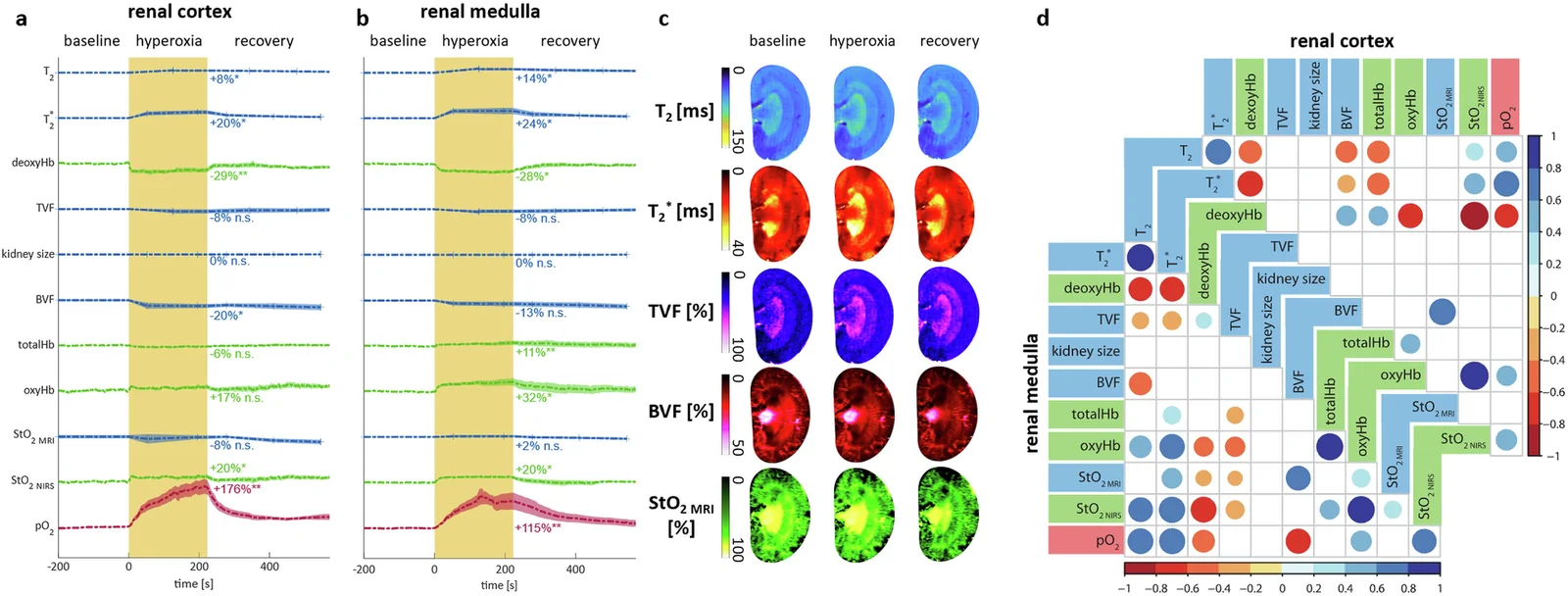
In vivo feasibility study—hyperoxia. Time courses of the parameters simultaneously acquired by the MRI-PHYSIOL-NIRS platform during hyperoxia (yellow shaded area) and recovery for **a** renal cortex and **b** renal medulla (blue = MRI-based measures, green = NIRS-based measures, red = PHYSIOL-based measure). Values are depicted as mean ± SEM (*n* = 11), scaled as percent change relative to baseline for each parameter separately. The numbers in the plots show the maximum percent change relative to the baseline (**p < 0.01, ***p < 0.001, non-parametric repeated-measures Friedman test followed by Dunn’s procedure). **c** Parametric maps from one representative rat, illustrating changes during the hyperoxia for T_2_^*^, T_2_, TVF, BVF and StO_2 MRI_, and the restoration of these MR measures during the recovery phase. **d** Matrices depicting repeated-measures correlation results among all parameters, for renal medulla and cortex separately. Color and size of the points are scaled to the correlation coefficient; only statistically significant correlations are depicted (repeated-measures correlation with Benjamini-Hochberg correction for multiple comparisons). The list of all correlation coefficients and *p*-values is provided in Supplementary Tables 1 and 2. All tests are two-sided.

Increasing the inspiratory O_2_ fraction is a widely used therapeutic intervention. Hyperoxia resulted in increased cortical and medullary pO_2_ (Fig. 5a, b) and in MRI-derived spatially resolved T_2_^*^, T_2_ (Fig. 5c), while BVF decreased. SO_2NIRS_ increased, while deoxyHb decreased (Fig. 5a). Upon the return to normoxia the parameters changed toward baseline levels (Fig. 5a–c). These results indicate that T_2_^*^, T_2_ changes during hyperoxia and recovery are essentially driven by tissue oxygenation, as shown by the significant correlations between pO_2_ and T_2_^*^, T_2_, and additionally supported by the correlations of T_2_^*^, T_2_ with StO_2 NIRS_ and deoxyHb (Fig. 5d, Table S1). The number of significant correlations among parameters during hyperoxia (Fig. 5d) was lower than during aortic occlusion (Fig. 4d). First, the changes in tissue pO_2_ are much higher than those in the parameters reflecting blood oxygenation, because O_2_ saturation of hemoglobin of arterial blood is almost 100% already during normoxia, and increases only marginally during hyperoxia in healthy subjects^31^. Second, TVF and kidney size did not change significantly during hyperoxia. Yet, significant changes in BVF during hyperoxia again had an impact on the pO_2_–T_2_^*^, T_2_ relationship, as indicated by the significant correlation of BVF with T_2_^*^, T_2_. This again underscores the necessity for MRI-based quantification of TVF and/or BVF together with kidney size for physiological interpretation of renal T_2_^*^, T_2_.

## Discussion

We introduce an integrated MRI-PHYSIOL-NIRS platform with unprecedented capabilities to comprehensively study renal oxygenation. We demonstrate in vivo that this technology enables specific and simultaneous assessment of non-invasive, spatially resolved MRI metrics and ground-truth PHYSIOL and NIRS metrics. By uniting these distinct yet complementary metrics, MRI-PHYSIOL-NIRS provides a unique opportunity to assess the links between MRI metrics and gold-standard oxygenation measures. This is evidenced by both experimental validation and by in vivo measurements at baseline and during two interventions that increased or decreased renal tissue oxygenation. We demonstrate successful integration of the three modalities in the MRI-PHYSIOL-NIRS platform and show that baseline results are consistent with data previously acquired in experiments using these modalities separately. The multimodal nature of the readouts, and the simultaneous measurements at high temporal resolution, combined with spatial mapping mark a substantive advance over prior studies using single modalities. The MRI-PHYSIOL-NIRS platform represents a major technical development to help decipher the complex relationship between true renal tissue oxygenation and apparent tissue oxygenation derived from MR oximetry. Our results show that TVF and BVF strongly influence T_2_ and T_2_^*^, and must be considered when interpreting these metrics. Their correlations with pO₂ indicate that structural and hemodynamic factors substantially affect apparent oxygenation, independent of true tissue oxygenation. Accounting for TVF and BVF is therefore essential for an accurate interpretation of renal MRI oximetry.

While previous studies have suggested that renal T_2_^*^, T_2_ are correlated with tissue pO_2_, very few have attempted to analyze their quantitative relationship^38^. These studies primarily relied on comparisons of MRI data with invasive pO_2_ measurements obtained in separate cohorts of animals^30,39,40^, in the left versus the right kidney^24^, or by sequential data acquired in the same animals at different times^41,42^. One study utilized a dedicated integrated MRI-PHYSIOL setup to obtain simultaneous data of both modalities from the same kidney in vivo^43^. That study found highly variable correlations between T_2_^*^, T_2_ and pO_2_, depending on the specific interventions performed, but did not obtain data on the major confounders of the T_2_^*^, T_2_ – pO_2_ relationship. The relationship between tissue pO_2_ and StO_2_ is much tighter than that between pO_2_ and the MRI metrics T_2_^*^, T_2_, and NIRS-derived renal regional StO_2_ has been used as a marker of oxygenation in clinical studies^44,45^. While simultaneous measurements of renal pO_2_ and NIRS-derived StO_2_ have been reported, no analyses of the quantitative relationship between these parameters have been published^31,36^. There are no reports on simultaneous measurements of renal T_2_^*^, T_2_ and NIRS-derived oxygenation data, however, one study found changes in NIRS- and MRI-derived cortical StO_2_ obtained in separate cohorts of rats well aligned^33^.

By linking MR oximetry to the ground truth metric pO_2_, we are closer to the ultimate objective of applying non-invasive MRI of renal oxygenation to patients in clinical practice. Stand-alone MRI oximetry could be implemented in various clinical settings, first of all as a tool for risk assessment and early diagnosis of AKI. Patients with pre-existing conditions such as CKD, diabetes, and old age are at increased risk for AKI in procedures such as transcutaneous cardiac interventions that utilize large volumes of x-ray contrast agents, and in surgical procedures that entail renal ischemia-reperfusion injury^9,10,46,47^. A case in point is kidney transplantation. In current clinical practice, the only kidney-related prognostic and diagnostic parameter in this context is eGFR^48–50^. The oxygenation status of donor kidneys could become a key prognostic criterion in the *Kidney Donor Profile Index*, which is used to determine the risk of graft failure. Measurement of oxygenation status might even expand the pool of available kidneys by its inclusion in the *Expanded Donor Criteria*^48–50^. For prospective living donors, an oximetry-based prognostic estimate of the future of their remaining kidney may help their decision-making. Finally, oximetry of both the recipient’s transplanted kidney and the donor’s remaining kidney may become a mainstay of non-invasive post-transplantation monitoring. We envision that stand-alone MR oximetry will become a clinical tool for diagnosis and risk stratification of AKI of origins other than ischemia/reperfusion. It may also prove beneficial for early diagnosis and for monitoring disease progression in various other renal disorders.

To achieve these goals, further research with interventions that emulate other clinical scenarios beyond aortic occlusion and hyperoxia are needed to fully elucidate the mechanisms underpinning the complex relationship between MRI-based apparent renal oxygenation and true tissue oxygenation^21,32,51,52^. In this way, we can assemble a “data dictionary” of changes in renal oxygenation in response to specific perturbations, and how MRI oximetry measurements relate to PHYSIOL and NIRS measurements. This can be used for exploratory data analysis to uncover the relationships among parameters. The MRI data can be fed into machine learning algorithms such as generative adversarial networks (GAN), with NIRS and PHYSIOL data serving as ground truth. Such GANs can be used for renal systems analysis, to evaluate how accurately true renal oxygenation can be predicted from MRI measures.

The next step is to extend MRI-PHYSIOL-NIRS analysis to pathological conditions. Our integrated platform could track oxygenation changes in various renal disease models. GANs trained on MRI-PHYSIOL-NIRS data could be used to distinguish mild, moderate, and severe pathology. Measuring animals with preconditioning or pre-existing pathology may reveal altered baseline values and subtle changes after subsequent interventions^52^. Longitudinal data could further train GANs to detect oxygenation changes before clinical signs appear, enabling early disease interception. The setup may also serve to evaluate novel therapies, with implications for clinical translation.

By combining MRI, NIRS and PHYSIOL probes in the same animal, MRI-PHYSIOL-NIRS supports the 3 R principles of animal research. If the three methods were performed as stand-alone experiments, substantially larger cohorts would be required to overcome biological variability among individual animals to achieve the same statistical power. MRI-PHYSIOL-NIRS can be used with longitudinal experimental designs where animals serve as their own controls, thus reducing animal numbers^53^. Furthermore, MRI-PHYSIOL-NIRS promotes refinement of animal studies: By calibrating MRI readouts against ground-truth measurements of tissue oxygenation, we generate a validated reference framework that will allow future studies to rely on MRI alone, without invasive probes, to refine pre-clinical kidney research.

The MRI-PHYSIOL-NIRS platform was implemented in a small-bore 9.4 T preclinical scanner rather than on a clinical human MR system. The high field strength was required to achieve sufficient sensitivity and spatial resolution for studies in small rodents, where organ size necessitates substantially smaller voxel volumes than in human imaging. In clinical MRI, the lower intrinsic sensitivity at 1.5–3.0 T is largely offset by the use of larger voxel sizes as seen in studies summarized in a recent review^5^. Importantly, the use of a preclinical model also enables invasive ground-truth measurements for calibration and validation of MRI-based readouts—procedures that would not be ethically feasible in human subjects. Together, this approach allows rigorous validation of MRI-derived physiological metrics under controlled experimental conditions.

The long-term translational goal is to establish MRI methods as reliable, non-invasive tools for measuring renal tissue oxygenation in both health and disease in patients. The value of the MRI-PHYSIOL-NIRS platform is its ability to calibrate MRI measurements with ground truth tissue oxygenation measurements. This approach enables systematic probing of renal oxygenation, investigations in pre-clinical models of renal disease, and responses to novel therapeutics, opening novel research territory into in vivo oxygenation phenotyping. While MRI-PHYSIOL-NIRS was customized to target the kidney, potential applications extend to other organs where changes in oxygenation have profound physiological consequences. Additional non-invasive modalities, such as photoacoustic imaging or MR thermometry, could enrich the methodological portfolio, and could be readily applied in humans.

In current routine clinical practice, physicians are completely lacking any reliable non-invasive means to observe oxygenation vulnerabilities that drive kidney pathology. The MRI-PHYSIOL-NIRS platform offers the tantalizing possibility of extending our understanding of renal (patho-)physiology from rodent models to humans and lays the foundation for future clinical applications to better diagnose and intercept kidney disease, where MRI oximetry could emerge as a standalone option empowered by artificial intelligence. This potential could have a significant impact on daily clinical practice.

## Methods

Implementation of MRI-PHYSIOL-NIRS presented critical challenges that required novel solutions and methodological breakthroughs on the biomedical engineering, optical imaging hardware, data acquisition, photon transport modeling and data analysis level. Practical impediments evoked by strong static magnetic fields used for MRI, by time varying magnetic field gradients used for spatial encoding in MRI, by space constraints of small bore MRI scanners that leave a clearance of only few centimeter, as well as potential electro-magnetic and acousto-mechanical interferences between the MRI equipment and the NIRS or PHYSIOL probes were addressed during the integration.

### Near infrared spectroscopy (NIRS) setup

For the assessment of deoxygenated (deoxyHb), oxygenated (oxyHb), total hemoglobin (totalHb) per tissue volume and O_2_ saturation of hemoglobin (StO_2 NIRS_) we implemented a NIRS setup tailored for the kidney. The concentration and O_2_ saturation of hemoglobin within the microvasculature was monitored with an in-house built continuous-wave NIRS setup. The setup was equipped with high degree of redundancy to minimize system failures. The diffuse reflectance of the incident light re-emerging from the kidney tissue at distances from the source between 1 mm and 8 mm was measured with a miniaturized fiber probe. The probe comprises 2 independent source fibers and 7 detection fibers (FG200LCC, 200 µm core diameter, numerical aperture (NA) 0.27, Thorlabs GmbH, Dachau, Germany) with a spacing of 1 mm. The diffuse transmittance through the kidney was measured with 3 additional detection fibers (FT200UMT, 200 µm core diameter, NA 0.39, Thorlabs GmbH) positioned at the opposite surface of the kidney with respect to the source/reflectance probe. Aluminum-coated reflective micro prisms (#66-771, Edmund Optics Ltd., York, UK) were attached to the ends of every individual fiber to guide the light into and out of the kidney, while maintaining a small profile in the small bore of the MRI magnet. Nine lasers (LightHub-6 & LightHub-4, 660, 685, 730, 785, 808, 850, 905, 980, and 1060 nm, Omicron-Laserage Laserprodukte GmbH, Rodgau, Germany) acting as light sources were sequentially switched on for 4.7 ms each in every acquisition cycle (50 ms). Using a 2 × 2 fiber switch (LEONI Fiber Optics GmbH, Neuhaus-Schierschnitz, Germany) this light was alternated between the two source fibers after every acquisition cycle. The light was detected with 10 avalanche photodiodes (APD, Hamamatsu Photonics Deutschland GmbH, Herrsching, Germany) equipped with reflective longpass-filters which reject all ambient light below 650 nm. Remaining background light was continuously monitored during the fiber switching time when all lasers were off, and was subtracted from all signals.

*From the biomedical engineering and optical imaging hardware perspective*, the small size of the rat kidney (long axis = 20.7 ± 0.7 mm, short axis = 12.7 ± 0.7 mm) and of the accessible MRI bore (diameter = 60 mm) given by the space used for the magnetic field gradient insert, the inner diameter of the radiofrequency (RF) antenna used for signal transmission and the surface RF coil used for reception, required highest priority and substantial miniaturization of the NIRS probes compared to conventional NIRS systems. The 90° deflection prisms at the fiber ends made it possible to guide the fibers in the confined space along the longitudinal axis of the MRI bore (Fig. 2a). The aluminum coating of the prisms ensures stable reflectivity even in mixed liquid/air surroundings. To collect the light by the reflectance probe preferably along the long axis of the kidney, which falls together with the longitudinal bore axis of the MRI scanner and the orientation of the optical fibers, the prisms were arranged in a staggered manner. Fiber and prism positioning was addressed by designing and manufacturing a scaffold that ensures alignment and stability of the probe head with a precision in the micrometer range. The prisms and fibers were glued into this scaffold (Fig. 2a). In this way, the challenge of aligning and safely connecting the micro prisms to the fibers could be solved. Furthermore, the prism size ensured that the aperture of the fibers could be fully utilized for the collection of light.

*For NIRS data acquisition* a high degree of redundancy was established to minimize failure rate. The use of nine lasers (nine different wavelengths) ensured immunity to single laser fluctuations. The two independent source fibers (labeled orange in Fig. 2a) helped to recognize and eliminate single fiber problems with optical contact, local tissue pigmentation, or even fiber fracture. Flexible optical fibers with a core diameter of 200 µm were chosen to avoid excess strain on the kidney and to fix them along the bore axis without strong forces needed for stiff fibers. The seven detection fibers of the reflectance probe allowed to collect spatially-resolved light re-emission from the kidney with 1 mm step size (Fig. 2a). This large number of thin fibers is superior to a lower number of thicker fibers in terms of information content, redundancy and flexibility of the probe. The three detection fibers placed on the dorsal surface of the kidney opposite to the light source fibers enable sensitivity to the medullary layer of the kidney. Furthermore, the combination of reflectance and transmittance measurements enhances discrimination between light absorption and light scattering.

For *NIRS signal detection* we addressed the challenges of the small size of the detection area of 0.0314mm^2^ by implementing sensitive APD. To protect the APDs from overexposure by ambient light, long pass filters were used to reject light below a wavelength of 650 nm. With this setup we achieved a median SNR of 100 (lognormally distributed over all channels with a multiplicative standard deviation of 3.0).

For *photon transport modeling*, the small size of the kidney and the very high absorption in the highly perfused renal tissue pose critical challenges. The diffusion approximation which is commonly applied to model photon transport is only valid for source-detector separations much larger than the inverse of the reduced scattering coefficient and for absorption coefficients two orders of magnitude below the reduced scattering coefficient. With a source-detector separation below 8 mm both requirements are not met at all for our NIRS probe head due to the severe constraints governed by the rat kidney anatomy. To overcome this limitation photon transport was modeled by full Monte Carlo simulation yielding photon path length histograms for all combinations of source detector separation, scattering coefficient and absorption coefficient. For this purpose, a GPU based Monte-Carlo simulation^54^ of the photon transport through the kidney was used to model the expected signals. The code was modified to produce histograms of the traveled path lengths of the photons in the medulla and the cortex separately. This allows the absorption value of the different tissue compartments to be taken into account in post processing. This procedure is called White Monte Carlo^55^ and largely reduces simulation time and noise of the produced probability distributions of photons re-emerging from the tissue at any given position.

For *NIRS data analysis*, the wavelength dependence of the scattering coefficients was modeled using a power law function. The absorption was modeled as a linear combination of the spectra of oxy- and deoxy-hemoglobin^56^, water^57^, fat^58^, and cytochrome C^59^. Since the contribution of fat to the absorption is only very small its concentration was fixed in the modeling to be half of the non-water content. The optical properties were extracted from the measured data using a generalized least-squares-fit of the database lookup to the data. The generalized least squares fit takes correlation of uncertainties of the observables into account by transforming the correlated observables into uncorrelated singular vectors. The correlations between the observables arise from the fact that the signals of all 9 wavelengths are detected with the same 10 detection fibers. Therefore, there is a correlation of the noise between all wavelengths on every single source-detector-pair and a correlation of the noise on every single wavelength over all detectors.

The reproducibility of the positioning and fixation of the NIRS probes with regard to the kidney surface is essential. However, fixation of the miniaturized NIRS probes is hardly reproducible on the microscopic scale due to biological variance. This constraint leads to fluctuations in the coupling efficiency of the light into the tissue and back into the detection fibers. The lasers show fluctuations in the coupling efficiency into the source fibers visible simultaneously in all detection fibers. These factors contribute additional correlated noise, which needed to be accounted for to extract reliable optical properties from the data. To quantify the noise level, structure and correlation, several calibration measurements were performed after the in vivo measurement using 11 distinct epoxy-resin and PDMS-based phantoms with known optical properties. The absorption coefficients of these calibration phantoms ranged from 0.08 to 1.6 cm^−1^ and the reduced scattering coefficients from 3 to 25 cm^−1^. These intervals are consistent with literature reports on optical properties of the rat kidney in the near-infrared^31,60^. The calibration procedure was repeated every day to account for deterioration of the probe heads. The acquired noise structure was used in the generalized least squares fit to harness the low noise level perpendicular to the correlated noise directions to get optimal estimates of the optical properties and consequently the hemoglobin concentration and its oxygen saturation in the renal cortex and medulla. Placing the probe head on the calibration phantoms covering a broad range of absorption/scattering coefficients and surface characteristics proved to be a suitable model for emulating variations in coupling uncertainties in the in vivo case. These uncertainties, however, still weaken the discrimination between absorption and scattering, leading to increased uncertainties in the absolute values of the hemoglobin concentrations. We mitigated this challenge partially by including them into the error model for determining the baseline optical properties of the rat kidney. Since the coupling remains stable during the intervention, the uncertainties were excluded when evaluating the temporal evolution of the properties. This stability results in higher precision for the estimated changes over time compared to the absolute baseline values. Occasionally, this method produced negative concentration estimates during the aortic occlusion when oxyhemoglobin concentration in the smaller vessels becomes negligible. In these cases, the higher precision of the changes was transferred to the absolute values by constraining the baseline concentrations to remain greater than or equal to the difference between the baseline and the lowest concentration point during the occlusion. Motion artifacts, which were observed in 5 animals, affected all wavelengths and detectors synchronously. They were corrected by including the patterns into the noise model.

### Setup of physiological probes (PHYSIOL)

The PHYSIOL setup established in physiology labs typically occupies a sphere of about 60 cm in diameter, including robotic arms to hold the probes. The MRI magnet bore has a clearance with a diameter of 20 cm. This rare commodity is further reduced by the gradient coils used for spatial encoding and magnetic field shimming and by the radiofrequency coils used for signal transmission. In our implementation, a linear polarized birdcage resonator was used for RF transmission in MRI that reduces the remaining space to a diameter of 72 mm. Further space is consumed by the RF coil used for MRI signal reception which was placed in a laterodorsal position as close as possible to the left kidney to achieve highest sensitivity for MRI. Our setup makes use of a four channel receive only rat RF coil tailored for abdominal imaging that reduces the clearance to a diameter of approximately 60 mm, which dictates the setup and positioning of the PHYSIOL probes (Fig. 2c). To meet the space constraints we followed our previously described approach^61^: For *local tissue oxygenation and local perfusion measurements* combined optical Laser-Doppler-flux and pO_2_ probes (pO_2_ E-Series Sensor, Oxford Optronix Ltd, Oxford, UK, Fig. 2b) attached to an OxyLite/OxyFlo-apparatus (Oxford Optronix) were used; for *blood pressure measurements* a catheter placed in the femoral artery together with a pressure transducer (DT-XX, Viggo-Spectramed, Swindon, UK) connected to an amplifier (TAM-A Plugsys Transducer, Hugo Sachs Elektronik - Harvard Apparatus GmbH, March, Germany) was used, and for *the measurement of total renal blood flow*, a perivascular flow probe (MC2PSB-MRI, Transonic Systems Inc., Ithaca, USA, Fig. 2b) connected to a flowmeter (TS420, Transonic Systems Inc.) was deployed. Data were monitored and recorded via HSE-HA HAEMODYN software (Hugo Sachs Elektronik - Harvard Apparatus GmbH) and analyzed with MATLAB (The MathWorks Inc., Natick, MA, USA). The in-house developed analysis script involved the extraction of heart and respiratory rates, of diastolic, systolic and mean arterial pressures, as well as synchronization of the PHYSIOL data with NIRS and MRI through a TTL pulse, and averaging over MRI acquisition time windows.

### MRI setup

PHYSIOL and NIRS were integrated in a 9.4 Tesla small bore MR system (Bruker Biospec 94/20, Bruker Biospin, Ettlingen, Germany). For MRI signal transmission a linear radiofrequency volume resonator (Bruker Biospin) was used. For MRI signal reception a 4-channel surface coil array tailored for rats (Bruker Biospin) was deployed^21^. Following T_2_-weighted pilot scans, selective shimming of magnetic field homogeneity on a volume of interest accommodating the left kidney was conducted. T_2_^*^ and T_2_ mapping were performed with respiratory-gated protocols. Details of the MR protocol including T_2_^*^ and T_2_ quantification and monitoring of kidney size are detailed in our previous work^21^. All experiments were repeated after the intravenous application of 2 mg Fe/kg BW ferumoxytol to measure the BVF and the O_2_ saturation of hemoglobin (StO_2 MRI_)^33^. The long component of a biexponential fit of the multi spin-echo signal decay was monitored as a surrogate of the TVF^32^. For assessment of the time course of the MRI metrics a region of interest (ROI) approach was used. Semi-automatic ROI placement was performed in accordance with our previous work^33^.

*Synchronization* of the MRI-PHYSIOL-NIRS modalities was achieved with a TTL pulse which was sent from the MRI scanner to the PHYSIOL and to the NIRS data acquisition boards including recording of time stamps.

### Animal preparation

**Fig. 6:**
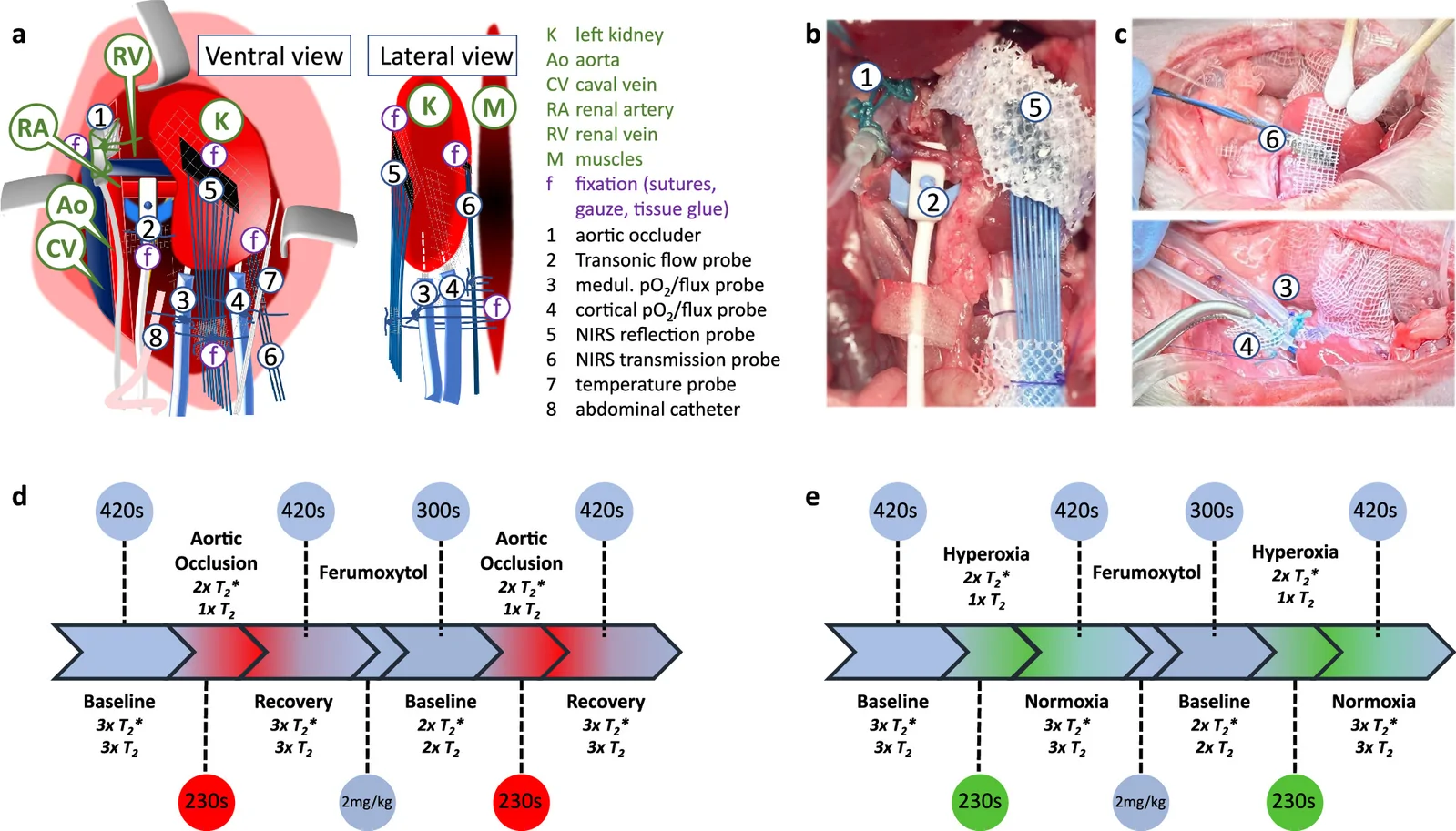
Animal preparation and experimental design. **a** Schematic view of the prepared anatomic structures, implanted probes and their fixation methods for the in vivo experiments in the MRI-PHYSIOL-NIRS platform comprising the ventral view to the area around the left kidney of a rat (left), the lateral view on the left kidney (middle), and the legend for letters and numbers used in the schematics and in (**b**, **c**) (right). **b**, **c** Photographs showing stages of the surgical preparation and implantation of the PHYSIOL and NIRS probes. **d** Schematic depicting the experimental protocol and timeline employed for the aortic occlusion study including the number of T_2_^*^ und T_2_ MRI scans acquired during the respective periods: Two cycles of the aortic occlusion-recovery intervention were performed, in between which ferumoxytol was injected to enable the MRI-based assessment of the renal blood volume fraction. **e** Schematic depicting the experimental protocol and timeline employed for the hyperoxia study including the number of T_2_^*^ und T_2_ MRI scans acquired during the respective periods: Two cycles of the hyperoxia-recovery intervention were performed, in between which ferumoxytol was injected to enable the MRI-based assessment of the renal blood volume fraction.

To offset the spatial constraints dictated by the MR environment relatively small rats were used. Experiments were performed in 25 male Wistar rats (aged 12–13 weeks; body mass 270–350 g; Envigo, Horst, The Netherlands). The animals had *ad libitum* access to standard diet and water and were housed under standard conditions with environmental enrichment. Rats were anesthetized with urethane (1.2 g/kg; Sigma-Aldrich, Steinheim, Germany) during surgery and MRI examinations. Urethane provides long-lasting anesthesia and has the least effects on cardiovascular and respiratory control compared to other anesthetics^62^. Rats underwent surgical procedures involving insertion of vascular catheters (carotid artery, jugular vein, femoral artery and vein) and invasive probes (flow probe around the left renal artery or vein and two combined pO_2_/flux probes, one inserted into the cortex, the other into the medulla of the left kidney) for quantitative measurements of renal hemodynamics and oxygenation, as previously described^61^. A catheter filled with saline to flush the abdomen and a temperature probe nearby the left kidney were placed loosely in the abdominal cavity (Fig. 6a, b).

For integration of MRI-PHYSIOL-NIRS we used a new approach for positioning of the cortical pO_2_/flux probe. Instead of advancing the probe from the caudal extent through the kidney to the cortical layer of the cranial extent as done in earlier studies^33^, it was inserted in a depth of approximately 1.5 mm in the caudal extent (Fig. 6a–c). The main reason to change the position of the cortical probe was that, in the earlier setup the longer puncture channel resulted in a larger volume of damaged tissue^3^, which had to be excluded from MRI analyses. The reflective prisms of the NIRS probes were positioned at the center of the dorsal surface of the kidney, for the reflection probes at the upper two-third of the ventral surface (Fig. 6b, c). The large distance to the flux probes (using 830 nm) ensured bias free measurements for both methods. The pO_2_ probes were working in a separate spectral range (~500 nm) with a filter excluding contamination by NIR light. All probes were fixed by gauze and tissue glue to the renal capsule and additionally by suture attached to the retroperitoneal muscles to prevent displacement (Fig. 6b, c).

For the in vivo feasibility study, surgical preparation and implementation of the PHYSIOL and NIRS probes was performed outside of the MR scanner room for safety reasons. With the surgical preparation and probes fixation completed, the rats were transferred into the MRI scanner. This transport of the rat required thorough practical considerations, since every movement of the animal after probe implantation might hold the risk for probe displacement. To fix and stabilize the position of the probes for a safe transport, and to meet the geometry constraints given by the MR setup, a dedicated animal holder was designed and built (Fig. 2c) using 3D CAD (Autodesk Inventor 2012, Autodesk Inc., San Rafael, USA) and rapid prototyping (BST 1200es, Alphacam GmbH, Schorndorf, Germany). The animal holder has a half pipe shape and accommodates the RF surface coil array used for MRI signal reception. For a seamless work flow the rat was placed on the holder in a supine position prior to surgery to ensure that it does not change its position until the end of the MRI-PHYSIOL-NIRS examinations. A bridge was positioned right behind the hindpaws of the rat with the goal to fixate the leads that connect the PHYSIOL and NIRS probe heads with the equipment positioned outside of the MR scanner room. The leads are semi-rigid so that any movement might disarrange the probes. For this reason, the bridge can be adjusted in height and distance to the animal. The animal holder in conjunction with the adjustable cable support enabled a safe transport of the rat into the MR scanner.

For positioning the animal holder in the iso-center of the MR system it was rotated by about 10-20° around the longitudinal axis to align the left kidney with the position of the receive RF coil. Respiration rate was monitored throughout the experiment using a small pad placed on the chest of the rat. This approach also served for triggering the MRI data acquisition^21^. For this purpose, the respiratory pad was placed such that during inspiration the pad is compressed. The pad was connected to a pressure transducer, the signal of which served to monitor the respiratory motion (P-resp, Model 1025, SA Instruments, New York, USA) and gate the MRI. The rats were spontaneously breathing and continuously provided with air (1 L/min) via a self-made respiratory mask positioned loosely in front of the nose so that exhalation is unhindered. Body temperature was maintained at 37 °C with a pad containing circulating warm water (Thermo Haake GmbH, Karlsruhe, Germany) connected to a water bath. Respiration, body temperature, arterial blood pressure, and heart rate were monitored continuously throughout the experiment^61^.

### Baseline

Before each intervention baseline measurements were obtained for 7 min (*n* = 25 rats). PHYSIOL and NIRS parameters were continuously measured, and MRI data were acquired three times with alternating T_2_^*^ and T_2_ scans, which also allow assessment of TVF, BVF, and StO_2 MRI_ (Fig. 6d, e).

### Acute pathological and therapeutic interventions

*Intrarenal hypoxia* was induced using aortic occlusion (*n* = 14 rats) inside of the MRI-PHYSIOL-NIRS platform. For this purpose, a dedicated MR-safe and remotely controlled inflatable occluder was positioned around the suprarenal aorta as part of the animal preparation as previously described (Fig. 6a, b)^61^. Inflation of the occluder results in complete closure of the aorta, and thereby, in intrarenal ischemia and hypoxia. Each occlusion lasted for 230 s (median) before the occluder was deflated to reestablish renal blood flow. Occlusion was verified by the absence of the flow-based blood signal in renal time-of-flight-MR angiography^21^. The duration of the interventions was a compromise between MR acquisition times and the reversibility of the intervention. It was necessary that the physiological state of the kidneys return to an undisturbed baseline function after the intervention, in order to repeat the same experiment a second time with the intravascular contrast agent ferumoxytol. This repetition allowed the estimation of the BVF. The recovery period comprised approximately 7 min. This was followed by intravenous injection of ferumoxytol (2 mg Fe/kg BW; Feraheme, AMAG Pharmaceuticals, Waltham, MA, USA)^33^, before a second cycle of the aortic occlusion—recovery intervention was performed (Fig. 6d).

*Hyperoxia* (*n* = 11 rats) was induced inside of the MRI-PHYSIOL-NIRS platform by increasing the fraction of inspired oxygen (FiO_2_) from 21% (normoxia) to 100% in the gas provided to the rat through the respiratory mask^61^. This results in hyperoxemia of the arterial blood. Hyperoxia lasted 230 s (median), before the FiO_2_ was restored to 21% (normoxia), followed by a recovery period of approximately 7 min. This was followed by intravenous injection of ferumoxytol (2 mg Fe/kg BW) before a second cycle of the hyperoxia—recovery intervention was performed (Fig. 6e).

### Statistical analysis

Changes in renal size, MRI, NIRS and physiological parameters in response to the aortic occlusion and hyperoxia interventions were analyzed using the non-parametric repeated-measures Friedman test, followed by the Dunn’s post-hoc test. Correlations between all parameters were assessed using repeated-measures correlation^63^, followed by correction for multiple comparisons using the Benjamini-Hochberg method. Data was analyzed using the statistical environment R v.3.6.3 with the packages “rstatix”, “dunn.test” and “rmcorr”. *P*-values < 0.05 were considered significant. All tests were two-sided.

### Ethics statement

All investigations comply with the ARRIVE guidelines and were approved by the Animal Welfare Department of Berlin’s State Office of Health and Social Affairs in accordance with the German Animal Protection Law and the Directive 2010/63/EU (permission reference: G0043/19).

## Supplementary information

Transparent Peer Review file

Supplementary Tables 1 and 2

## Data Availability

The collected experimental data that support the findings of this study are available as tables on GitHub at: https://github.com/ThomasGladytz/MRI-PHYSIOL-NIRS^64^. The complete raw data is available upon request from the corresponding author.

## Code availability

The data analysis code used in this study was mainly written in MATLAB version R2021a and R2025b. It is available on GitHub at: https://github.com/ThomasGladytz/MRI-PHYSIOL-NIRS^64^.
