# Supplementary material for: MRI-PHYSIOL-NIRS: an integrated platform to study kidney oxygenation
Source: Commun Eng. 2026 Oct 16;5:176. doi: 10.1038/s44172-026-00799-x (PMC13675240; doi:10.1038/s44172-026-00799-x)
Supplement: Supplementary file 1 [file 44172_2026_799_MOESM1_ESM.pdf]

nature portfolio

Peer Review File

MRI-PHYSIOL-NIRS: An Integrated Platform to Study Kidney Oxygenation

Corresponding Author: Dr Thomas Gladytz

This file contains all reviewer reports in order by version, followed by all author rebuttals in order by version.

Version 0:

Reviewer comments:

Reviewer #1

(Remarks to the Author)
This manuscript describes the development and in vivo validation of a multimodal MRI-PHYSIOL-NIRS platform integrating renal MRI oximetry (T2 and T2*) with invasive gold-standard measurements of tissue pO_2 and complementary NIRS-derived hemodynamic parameters in a rat model. The study addresses an important gap in the field, namely the lack of direct validation of MRI-derived surrogate markers of renal oxygenation against quantitative tissue oxygen measurements acquired simultaneously in the same subject. The use of controlled hypoxic (aortic occlusion) and hyperoxic interventions provides a robust framework to interrogate the relationship between MRI metrics, physiological parameters, and true tissue oxygenation in both cortex and medulla. Overall, the work represents a technically sophisticated and conceptually relevant contribution to renal oxygenation research.

The translational perspective could be strengthened. It would be helpful to clarify how this platform, or its MRI component alone after calibration, could realistically be implemented in clinical settings. In particular, the authors should outline which patient populations (e.g., AKI, early CKD, diabetic nephropathy, perioperative ischemia-reperfusion settings) would most likely benefit from renal oxygenation monitoring, and at what disease stage this technique would be most informative.

The introduction provides a clear and well-structured overview of renal oxygenation physiology and the limitations of current biomarkers. The positioning of tissue pO_2 as the gold standard is appropriate.

Line 59: A citation supporting the statement regarding the prevalence of AKI patients progressing to CKD is required.

Line 63: The authors may wish to explicitly emphasize the potential of renal oxygenation as an early prognostic biomarker, particularly in defined patient groups such as early CKD, diabetic kidney disease, or patients at high risk for ischemic injury.

Line 76: The sentence is partially repetitive of the preceding statement and could be streamlined.

Line 80: The limited clinical adoption of renal MRI oximetry may not solely reflect mechanistic uncertainty. Practical constraints, including scanner availability, cost, and the current adequacy of serum creatinine and UACR for late-stage disease monitoring, should be discussed as additional barriers to clinical implementation.

Line 124: The abbreviation “ADC board” should be defined at first mention.

Lines 211 and 232: The data convincingly demonstrate the importance of quantifying tubular volume fraction (TVF) and blood volume fraction (BVF) when interpreting T2 and T2* changes. The correlations with pO_2 underscore that these structural and hemodynamic confounders substantially influence apparent oxygenation measures. This is a key mechanistic insight of the study.

Line 258: The authors provide an appropriate reflection on limitations of prior studies and clearly articulate how the present work addresses these shortcomings through simultaneous multimodal acquisition.

Line 297: Given the use of a 9.4T preclinical system, the authors should discuss in more detail the expected reproducibility and sensitivity of these findings at clinical field strengths (1.5–3T). Differences in susceptibility effects, SNR, and temporal resolution may affect the generalizability of T2* and T2 calibration to clinical scanners.

Line 426: Are the absorption coefficients of the calibration phantoms within physiologically relevant ranges for renal tissue? If so, appropriate references should be provided.

Line 435: The decision to exclude coupling uncertainties during temporal evaluation assumes stability of probe positioning. The authors should provide quantitative justification for this assumption, as residual calibration drift could bias dynamic estimates.

Line 437: Negative concentration estimates were occasionally observed. The underlying causes (e.g., model instability, ill-conditioned fitting, noise correlations) should be discussed in more detail.

The potential contribution of systemic hematological parameters (e.g., serum hemoglobin, hematocrit) to variability in T2, T2*, and pO_2 measurements should also be considered, particularly given their influence on blood oxygen-carrying capacity and susceptibility effects.

Line 509: The distance between NIRS and PHYSIOL probes is described as maximal, but inter-animal variability in this distance may influence signal contamination or spatial sampling bias. Clarification of the variability range and its potential impact would be valuable.

The choice of 230 seconds for hypoxia and hyperoxia induction should be justified. Was this duration derived from prior literature, pilot data, or constrained by MRI acquisition timing?

Table 1: It would be useful to clarify whether compartment-specific cross-sectional area measurements (cortex versus medulla) are feasible and reproducible. Additionally, the apparent inverse trend in StO_2 between cortex and medulla across modalities (MRI vs. NIRS) warrants further discussion regarding spatial sensitivity and compartment weighting of each technique.

Figure 1: Additional confounders mentioned in the legend are not clearly visible in the schematic and should be incorporated more explicitly into the figure.

Figure 2: The NIRS system and PHYSIOL rack could be more clearly labeled to aid readers less familiar with the technical setup.

Figure 3: The varying y-axis scales across panels limit direct visual comparison of relative changes. Presentation of normalized values with consistent scaling, or inclusion of mean ± SD summary plots, may improve interpretability.

Figures 4 and 5: These figures effectively illustrate the differential behavior of MRI-derived parameters under hypoxic and hyperoxic conditions and represent a strong component of the manuscript.

Figure 6: Panels d and e could potentially be consolidated, as the principal difference lies in the type of intervention rather than experimental structure.

This study provides a rigorous and technically advanced validation of renal MRI oximetry against invasive gold-standard measurements of tissue pO_2 within a unified multimodal framework. By systematically dissecting the influence of structural and hemodynamic confounders, the authors clarify the mechanistic underpinnings of T2 and T2* as surrogate markers of renal oxygenation. The work substantially advances the methodological foundation required for translational application of renal MRI oximetry. Further discussion of clinical implementation and field-strength dependence would strengthen its translational impact.

Reviewer #2

(Remarks to the Author)
I co-reviewed this manuscript with one of the reviewers who provided the listed reports. This is part of the Communications Engineering initiative to facilitate training in peer review and to provide appropriate recognition for Early Career Researchers who co-review manuscripts.

Reviewer #3

(Remarks to the Author)
Major claims of the paper:

The paper explores a novel platform (MRI-PHYSIOL-NIRS) designed to link non-invasive MRI-measurements of

oxygenation to invasive oxygenation measurements by Near-Infrared Spectoscophy (NIRS)and gold standard pO2-probes. Non-invasive reliable O2 measurements by MRI might potentially be of great clinical importance since the kidneys are vulnerable to hypoxia. This could be a powerful tool to (early) detection of disease and to follow therapeutic interventions.

In the study, the authors have applied an advanced experimental set-up and studied correlations between multiple oxygen-related parameters (T2*, T2, pO2, StO2NIRS, StO2MRI, TVF, BVF and kidney size) both under physiological conditions and under pathophysiological conditions (hypoxia/hyperoxia).

Only small intra-individual fluctuations for all measured parameters were found speaking for robust measurements that were found to be in line with litterature.

Clamping the aorta above the renal arteries caused significant changes in almost all measured parameters. After release of the clamp, most parameters returned to pre-clamp levels within a few minutes. Significant correlations were found between pO2 and T2* and T2. Significant correlations were also found among almost all oxygen related parameters for both cortex and medulla.
Correlations of T2* and T2 to TVF, BVF and kidney size indicate that TVF and BVF have considerable inpact on the relationship between true and apparent oxygenation. Thus, physiological interpretation of T2* and T2 as markers for renal tissue oxygenation must account for changes in these variables. MRI-based quantification of TVF/BVF/kidney size should be performed for physiological interpretation of renal T2* and T2.

Basically the same goes for hyperoxia even though the changes were not so clear since no mechanical intervention was made.

The MRI-PHYSIOL-NIRS platform provides a solid setup for studies of different pathological renal conditions as well as for evaluation of (new) therapies. More studies with calibration work using machine learning remains to be done before MRI can be used as a single method for quantification of renal oxygenation.

The setup is novel and technically advanced and of interest for future renal research.
The work is convincing and I believe that this new platform will influence thinking in the field.

In the setup, the authors have cared for trying to minimize possible interactions between the different modalities used.

The authors discuss the fact that the high MRI field strength (9,4T) of the MRI scanner used in the study provides greater sensitivity and better temporal resolution compared to clinical scanners of typically 1,5 - 3T.

The study seems well executed and the manuscript is well written. I have no major or minor concerns.

Version 1:

Reviewer comments:

Reviewer #1

(Remarks to the Author)
MRI-PHYSIOL-NIRS review-REVISIONS, Nature Communications Engineering Begawan Siadari, b.a.siadari@lumc.nl

Abstract:

· Overall abstract substantially reflected the research gap (lack of direct comparison of renal oxygenations with T2*) and methodology (MRI-NIRS-PHSYIOL) of this study.

Introduction:

· All previous suggestions have been integrated into the latest version of manuscript.

· Clinical translation and importance have been highlighted substantially as suggested.

Results:

· Previous missing abbreviation has been added as suggested.

· With all the abbreviations of renal oxygenation parametrics, consider providing a table that explicitly mentions all parameters.

Discussion:

· Emphasized the importance of TVF and BVF in renal oxygenations parameters.

· Line 232: Great way of highlighting the need of renal functions assessment in clinical settings.

o Consider add inghow this technique could also be utilize for other renal diseases progressions and risk stratifications, maybe the use expands beyond that for renal transplantation.

· Line 297: 9.4T -> clinical scanner (1.5 or 3T): consider adding citations for similar studies, both in-vivo and preclinical, on renal oxygenation done before.

o Consider expanding on highlighting the difficulty of clinical translations and novelty of this study in direct renal oxygenations comparisons.

Method:

· Absorption coefficient remarks have been answered.

· Remarks on coupling uncertainties was explained in rebuttal letter and manuscript.

· Line 437: What would cause the difference in baseline concentrations? Since these negative concentrations were only observed in some of the measurements.

o Could these negative concentrations be caused by using anaesthesia or probe introduction (methodological) or physiological variation in the mice model?

· Line 509: clarified the use of maximal distance between NIRS and PHYSIOL probes.

· The choice of occlusion time (230 sec) was clarified methodologically.

Reviewer #2

(Remarks to the Author)
I co-reviewed this manuscript with one of the reviewers who provided the listed reports. This is part of the Communications Engineering initiative to facilitate training in peer review and to provide appropriate recognition for Early Career Researchers who co-review manuscripts.

Open Access This Peer Review File is licensed under a Creative Commons Attribution 4.0 International License, which permits use, sharing, adaptation, distribution and reproduction in any medium or format, as long as you give appropriate credit to the original author(s) and the source, provide a link to the Creative Commons license, and indicate if changes were made.
In cases where reviewers are anonymous, credit should be given to 'Anonymous Referee' and the source.
The images or other third party material in this Peer Review File are included in the article’s Creative Commons license, unless indicated otherwise in a credit line to the material. If material is not included in the article’s Creative Commons

license and your intended use is not permitted by statutory regulation or exceeds the permitted use, you will need to obtain permission directly from the copyright holder.
To view a copy of this license, visit https://creativecommons.org/licenses/by/4.0/

We thank the editor and reviewers for their time and constructive feedback on our manuscript. We have carefully considered the comments and have revised the manuscript accordingly. We have shortened the abstract to keep the limit of 150 words. Below we provide a point-by-point response to each comment.

Reviewer #1 (Remarks to the Author):

This manuscript describes the development and in vivo validation of a multimodal MRI-PHYSIOL-NIRS platform integrating renal MRI oximetry (T2 and T2*) with invasive gold-standard measurements of tissue pO_2 and complementary NIRS-derived hemodynamic parameters in a rat model. The study addresses an important gap in the field, namely the lack of direct validation of MRI-derived surrogate markers of renal oxygenation against quantitative tissue oxygen measurements acquired simultaneously in the same subject. The use of controlled hypoxic (aortic occlusion) and hyperoxic interventions provides a robust framework to interrogate the relationship between MRI metrics, physiological parameters, and true tissue oxygenation in both cortex and medulla. Overall, the work represents a technically sophisticated and conceptually relevant contribution to renal oxygenation research.

The **translational perspective** could be strengthened. It would be helpful to clarify how this platform, or its MRI component alone after calibration, could realistically be implemented in clinical settings. In particular, the authors should outline which patient populations (e.g., AKI, early CKD, diabetic nephropathy, perioperative ischemia-reperfusion settings) would most likely benefit from renal oxygenation monitoring, and at what disease stage this technique would be most informative.

>>>> We thank the reviewer for their encouraging feedback and suggestions for improvement. We agree that the translational aspect of our work can be highlighted more rigorously. Following the reviewer's suggestions, we added the following paragraph to the Discussion section:

"Stand-alone MRI oximetry could be implemented in various clinical settings, first of all as a tool for risk assessment and early diagnosis of AKI. Patients with pre-existing conditions such as CKD, diabetes, and old age are at increased risk for AKI in procedures such as transcutaneous cardiac interventions that utilize large volumes of x-ray contrast agents, and in surgical procedures that entail renal ischemia-reperfusion injury.[9,10,44,45] *A case in point is kidney transplantation. In current clinical practice, the only kidney-related prognostic and diagnostic parameter in this context is eGFR.*[46-48] *The oxygenation status of donor kidneys could become a key prognostic criterion in the Kidney Donor Profile Index, which is used to determine the risk of graft failure. Measurement of oxygenation status might even expand the pool of available kidneys by its inclusion in the Expanded Donor Criteria.*[46-48] *For prospective living donors, an oximetry-based prognostic estimate of the future of their remaining kidney may help their decision-making. Finally, oximetry of both the recipient's transplanted kidney and the donor's remaining kidney may become a mainstay of non-invasive post-transplantation monitoring."*

The introduction provides a clear and well-structured overview of renal oxygenation physiology and the limitations of current biomarkers. The positioning of tissue pO_2 as the gold standard is appropriate.

Line 59: A citation supporting the statement regarding the prevalence of AKI patients progressing to CKD is required.
>>>> Following the reviewer’s recommendation additional references were added to the manuscript.

Chawla LS, Eggers PW, Star RA, Kimmel PL. Acute kidney injury and chronic kidney disease as interconnected syndromes. NEnglJMed. 2014;371(1):58–66 DOI: 10.1056/NEJMra1214243.

Goldstein SL, Jaber BL, Faubel S, Chawla LS. AKI transition of care: a potential opportunity to detect and prevent CKD. ClinJAmSocNephrol. 2013;8(3):476–83 DOI: 10.2215/CJN.12101112

Line 63: The authors may wish to explicitly emphasize the potential of renal oxygenation as an early prognostic biomarker, particularly in defined patient groups such as early CKD, diabetic kidney disease, or patients at high risk for ischemic injury.

>>>> Following the reviewer’s suggestion additional text was added to the introduction section to emphasize the potential of renal oxygenation as an early diagnostic and prognostic marker:

“Given the vital role of oxygenation in kidney pathology, renal oxygenation has strong potential as an early diagnostic biomarker in AKI — where it may enable detection of injury before irreversible functional decline occurs — as well as a prognostic biomarker, particularly in patients at increased risk of ischemic injury, patients with early CKD or early diabetic kidney disease. Current clinical practice, however, falls short of this goal.”

Line 76: The sentence is partially repetitive of the preceding statement and could be streamlined.

>>>>> The paragraph was streamlined and now reads:

“Magnetic resonance imaging (MRI) is a cornerstone of diagnostic imaging, offering non-invasive, high-resolution assessment of anatomy with whole-organ coverage. It can also probe tissue oxygenation in vivo, as deoxygenated hemoglobin (deoxyHb) is paramagnetic and alters MRI signals. Accordingly, transverse relaxation times T_2 and T_2^* are sensitive to deoxyHb levels and are used as indirect markers of tissue oxygenation. However, the relationship of these metrics with true tissue oxygenation (pO_2) is confounded by several physiological factors including renal blood and tubular volume changes that affect kidney size and signal interpretation (Figure 1a).”

Line 80: The limited clinical adoption of renal MRI oximetry may not solely reflect mechanistic uncertainty. Practical constraints, including scanner availability, cost, and the current adequacy of serum creatinine and UACR for late-stage disease monitoring, should be discussed as additional barriers to clinical implementation.

>>>>> Following the reviewer’s advice this paragraph has been carefully revised to include some of the practical constraints of clinical adoption of MRI oximetry:

“Clear mechanistic links between MRI metrics of apparent oxygenation and true tissue oxygenation remain lacking. Aside from practical constraints including scanner availability and costs, these discrepancies are a major reason why clinical use of MRI for renal oximetry has been limited.”

Line 124: The abbreviation “ADC board” should be defined at first mention.

>>>>> The abbreviation ADC is now defined as “analog-to-digital converter”.

Lines 211 and 232: The data convincingly demonstrate the importance of quantifying tubular volume fraction (TVF) and blood volume fraction (BVF) when interpreting T2 and T2* changes. The correlations with pO_2 underscore that these structural and hemodynamic confounders substantially influence apparent oxygenation measures. This is a key mechanistic insight of the study.

>>>>> We highlighted this key insight in the discussion again:
“Our results show that tubular volume fraction (TVF) and blood volume fraction (BVF) strongly influence T_2 and T_2, and must be considered when interpreting these metrics. Their correlations with pO_2 indicate that structural and hemodynamic factors substantially affect apparent oxygenation, independent of true tissue oxygenation. Accounting for TVF and BVF is therefore essential for an accurate interpretation of renal MRI oximetry.”*

Line 258: The authors provide an appropriate reflection on limitations of prior studies and clearly articulate how the present work addresses these shortcomings through simultaneous multimodal acquisition.

>>>>> We value the reviewer’s appreciation of our work.

Line 297: Given the use of a 9.4T preclinical system, the authors should discuss in more detail the expected reproducibility and sensitivity of these findings at clinical field strengths (1.5–3T). Differences in susceptibility effects, SNR, and temporal resolution may affect the generalizability of T2* and T2 calibration to clinical scanners.

>>>>> Upon careful reflection we revised this paragraph to elaborate on the expected reproducibility and sensitivity of our findings at clinical field strengths:

“The MRI-PHYSIOL-NIRS platform was implemented on a small-bore 9.4 T preclinical scanner rather than a clinical human MR system. The high field strength was required to achieve sufficient sensitivity and spatial resolution for studies in small rodents, where organ size necessitates substantially smaller voxel volumes than in human imaging. In clinical MRI, the lower intrinsic sensitivity at 1.5–3.0 T is largely offset by the use of larger voxel sizes. Importantly, the use of a preclinical model also enables invasive ground-truth measurements for calibration and validation of MRI-based readouts—procedures that would not be ethically feasible in human subjects. Together, this approach allows rigorous validation of MRI-derived physiological metrics under controlled experimental conditions.”

Line 426: Are the absorption coefficients of the calibration phantoms within physiologically relevant ranges for renal tissue? If so, appropriate references should be provided.

>>>> To address the reviewer's suggestion the text has been revised and an extra reference has been inserted.

"The absorption coefficients of these calibration phantoms ranged from 0.08 to 1.6 cm^{-1} and the reduced scattering coefficients from 3 to 25 cm^{-1}. These intervals are consistent with literature reports on optical properties of the rat kidney in the near-infrared.[31,60]*."*

Line 435: The decision to exclude coupling uncertainties during temporal evaluation assumes stability of probe positioning. The authors should provide quantitative justification for this assumption, as residual calibration drift could bias dynamic estimates.

>>>>> Since the calibrations were carried out over a duration of 30 minutes, long and medium-term drifts of the system are covered by the calibration procedure, and are therefore already incorporated into the noise model. Special treatment was required in case of motion artifacts which occurred in 5 cases. The motion events were easily discernible from physiological changes by their spectral and distance dependence. These probe motion events typically affect all wavelengths and detectors synchronously by a similar amount. They were removed by specifically including those patterns into the noise model. To describe this procedure, the following sentence was added to the manuscript:

"Motion artifacts, which were observed in 5 animals, affected all wavelengths and detectors synchronously. They were corrected by including the patterns into the noise model."

Line 437: Negative concentration estimates were occasionally observed. The underlying causes (e.g., model instability, ill-conditioned fitting, noise correlations) should be discussed in more detail.

>>>>> Thank you for this valuable comment. We confirm that the aortic occlusion intervention depletes the oxyhemoglobin concentration in the smaller vessels (which NIRS is preferentially sensitive to) down to negligible values. If the true value is essentially 0 all unconstrained estimates, that inherently have errors, will yield negative results in almost 50% of the cases. For more clarity in that respect the respective sentence was modified to read:
"Occasionally, this method produced negative concentration estimates during the aortic occlusion when oxyhemoglobin concentration in the smaller vessels becomes negligible. In these cases, the higher precision of the changes was transferred to the absolute values by constraining the baseline concentrations to remain greater than or equal to the difference between the baseline and the lowest concentration point during the occlusion."

The potential contribution of systemic hematological parameters (e.g., serum hemoglobin, hematocrit) to variability in T2, T2*, and pO_2 measurements should also be considered, particularly given their influence on blood oxygen-carrying capacity and susceptibility effects.

>>>> We confirm that the Introduction section and the legend of Figure 1A read:

"*However, the relationship of these metrics with true tissue oxygenation (pO_2) depends on factors such as blood oxygen pressure, O_2 saturation of hemoglobin (StO_2), hematocrit, and on the quantity of deoxyHb within an image voxel (Figure 1a).*" and in the Legend to Fig. 1a: *"Additional confounders include hematocrit, the oxyHb dissociation curve, and O_2 diffusion from blood into tissue."*
For the short timescale of the interventions discussed here, the changes in the hematological parameters are negligible. Therefore, we did not include them in the discussion of the current manuscript. We thank the reviewer for the reminder to consider them in future work, with interventions where the hematological parameters are changed.

Line 509: The distance between NIRS and PHYSIOL probes is described as maximal, but inter-animal variability in this distance may influence signal contamination or spatial sampling bias. Clarification of the variability range and its potential impact would be valuable.

>>>>> The large distance between NIRS and flux probes ensured that signal contamination was negligible. Generally, the flux/pO2 probe regions were excluded from the NIRS and MR sampling area as the needle insertion inherently induces lesions, that interfere with the evaluation of the state of the kidney. To underline the contamination free measurements, we changed the text on the NIRS probe placement to:

"The reflective prisms of the NIRS transmission probes were positioned at the center of the dorsal surface of the kidney, for the reflection probes at the upper two-third of the ventral surface (Figure 6b,c). The large distance to the flux probes (using 830 nm) ensured bias free measurements for both methods. The pO2 probes were working in a separate spectral range (~500 nm) with a filter excluding contamination by NIR light."

The choice of 230 seconds for hypoxia and hyperoxia induction should be justified. Was this duration derived from prior literature, pilot data, or constrained by MRI acquisition timing?

>>>>> The following text was added to the respective methods section to clarify the choice of the intervention timing:
"The duration of the interventions was a compromise between MR acquisition times and the reversibility of the intervention. It was necessary that the physiological state of the kidneys return to an undisturbed baseline function after the intervention, in order to repeat the same experiment a second time with the intravascular contrast agent ferumoxytol. This repetition allowed the estimation of the blood volume fraction."

Table 1: It would be useful to clarify whether compartment-specific cross-sectional area measurements (cortex versus medulla) are feasible and reproducible. Additionally, the apparent inverse trend in StO_2 between cortex and medulla across modalities (MRI vs. NIRS) warrants further discussion regarding spatial sensitivity and compartment weighting of each technique.

>>>>> The contrast between the renal cortex and the outermost layer of the medulla is very subtle in the MR images. Only inner-medullary stratification could be feasible, but was not the focus of this

work. Therefore, we did not differentiate the sizes here. Nevertheless, we thank the reviewer for the suggestion, and will consider this aspect in future work. The different trends of the StO_2 and especially the BVF and total hemoglobin concentration, are indeed interesting observations. As suggested by the reviewer, they most likely are due to the different spatial sensitivity and compartment weighting of each technique. We will investigate this difference further in our future work.

Figure 1: Additional confounders mentioned in the legend are not clearly visible in the schematic and should be incorporated more explicitly into the figure.

>>>>> Figure 1a includes "**totalHb / blood volume**" as a confounder; while the Legend reads "Additional confounders include **hematocrit**, the oxyHb dissociation curve, and O_2 diffusion from blood into tissue." Unfortunately, we did not point out that total Hb per blood volume is essentially equivalent with hematocrit. We revised the relevant sentence in the Legend, which now reads

"*Additional confounders include totalHb / blood volume (equivalent with hematocrit), the oxyHb dissociation curve, and O_2 diffusion from blood into tissue.*

Figure 2: The NIRS system and PHYSIOL rack could be more clearly labeled to aid readers less familiar with the technical setup.

>>>>> We thank the reviewer for this suggestion and have added labels as proposed.

Figure 3: The varying y-axis scales across panels limit direct visual comparison of relative changes. Presentation of normalized values with consistent scaling, or inclusion of mean ± SD summary plots, may improve interpretability.

>>>>> We have incorporated the Reviewer's suggestion to show the mean values (black line) in the revised version of Figure 3a. The purpose of the figure is not to compare values between cortex and medulla, and not to compare values of one parameter with another. Rather, the purpose of the figure is to highlight the consistency of the values over the three baseline measurements for each parameter, and therefore we consider that it is necessary to use the full dynamic range, and to present the data as real values in the original units, rather than normalized values. We hope that inclusion of the mean values improves the clarity of the figure; as we show data from each individual animal, the reader can appreciate the distribution directly from the data, and therefore plotting the standard deviation is not necessary.

Figures 4 and 5: These figures effectively illustrate the differential behavior of MRI-derived parameters under hypoxic and hyperoxic conditions and represent a strong component of the manuscript.

>>>>> We thank the reviewer for the encouraging feedback.

Figure 6: Panels d and e could potentially be consolidated, as the principal difference lies in the type

of intervention rather than experimental structure.

>>>>> We thank the reviewer for this comment. We changed the coloring of the panels to better communicate their difference. We want to keep separate panels to communicate, that these experiments were done independently, and on different animals.

This study provides a rigorous and technically advanced validation of renal MRI oximetry against invasive gold-standard measurements of tissue pO_2 within a unified multimodal framework. By systematically dissecting the influence of structural and hemodynamic confounders, the authors clarify the mechanistic underpinnings of T2 and T2* as surrogate markers of renal oxygenation. The work substantially advances the methodological foundation required for translational application of renal MRI oximetry. Further discussion of clinical implementation and field-strength dependence would strengthen its translational impact.

Reviewer #2 (Remarks to the Author):

I co-reviewed this manuscript with one of the reviewers who provided the listed reports. This is part of the Communications Engineering initiative to facilitate training in peer review and to provide appropriate recognition for Early Career Researchers who co-review manuscripts.
>>>>> We thank reviewer 2 for the contributions to the review process.

Reviewer #3 (Remarks to the Author):

Major claims of the paper:

The paper explores a novel platform (MRI-PHYSIOL-NIRS) designed to link non-invasive MRI-measurements of oxygenation to invasive oxygenation measurements by Near-Infrared Spectoscophy (NIRS)and gold standard pO2-probes. Non-invasive reliable O2 measurements by MRI might potentially be of great clinical importance since the kidneys are vulnerable to hypoxia. This could be a powerful tool to (early) detection of disease and to follow therapeutic interventions.

In the study, the authors have applied an advanced experimental set-up and studied correlations between multiple oxygen-related parameters (T2*, T2, pO2, StO2NIRS, StO2MRI, TVF, BVF and kidney size) both under physiological conditions and under pathophysiological conditions (hypoxia/hyperoxia).

Only small intra-individual fluctuations for all measured parameters were found speaking for robust measurements that were found to be in line with literature.

Clamping the aorta above the renal arteries caused significant changes in almost all measured parameters. After release of the clamp, most parameters returned to pre-clamp levels within a few minutes. Significant correlations were found between pO2 and T2* and T2. Significant correlations

were also found among almost all oxygen related parameters for both cortex and medulla. Correlations of T2* and T2 to TVF, BVF and kidney size indicate that TVF and BVF have considerable inpact on the relationship between true and apparent oxygenation. Thus, physiological interpretation of T2* and T2 as markers for renal tissue oxygenation must account for changes in these variables. MRI-based quantification of TVF/BVF/kidney size should be performed for physiological interpretation of renal T2* and T2.

Basically the same goes for hyperoxia even though the changes were not so clear since no mechanical intervention was made.

The MRI-PHYSIOL-NIRS platform provides a solid setup for studies of different pathological renal conditions as well as for evaluation of (new) therapies. More studies with calibration work using machine learning remains to be done before MRI can be used as a single method for quantification of renal oxygenation.

The setup is novel and technically advanced and of interest for future renal research.
The work is convincing and I believe that this new platform will influence thinking in the field.

In the setup, the authors have cared for trying to minimize possible interactions between the different modalities used.

The authors discuss the fact that the high MRI field strength (9,4T) of the MRI scanner used in the study provides greater sensitivity and better temporal resolution compared to clinical scanners of typically 1,5 - 3T.

The study seems well executed and the manuscript is well written. I have no major or minor concerns.

>>>>> We thank reviewer 3 for their review and the encouraging statements.

EDITOR

1. With all the abbreviations of renal oxygenation parametrics, consider providing a table that explicitly mentions all parameters.
2. Consider adding how this technique could also be utilised for other renal diseases progressions and risk stratifications - maybe the use expands beyond that for renal transplantation.
3. Line 297: 9.4T -> clinical scanner (1.5 or 3T): consider adding citations for similar studies, both in-vivo and preclinical, on renal oxygenation done before.
4. Consider expanding on highlighting the difficulty of clinical translations and novelty of this study in direct renal oxygenations comparisons.
5. Line 437: What would cause the difference in baseline concentrations? Since these negative concentrations were only observed in some of the measurements.
6. Could these negative concentrations be caused by using anaesthesia or probe introduction (methodological) or physiological variation in the mice model?

REV #1

Abstract:

Overall abstract substantially reflected the research gap (lack of direct comparison of renal oxygenations with T2*) and methodology (MRI-NIRS-PHSYIOL) of this study.

Introduction:

All previous suggestions have been integrated into the latest version of manuscript.

Clinical translation and importance have been highlighted substantially as suggested

Results:

Previous missing abbreviation has been added as suggested.

With all the abbreviations of renal oxygenation parametrics, consider providing a table that explicitly mentions all parameters.

>>>>> The abbreviations table is supplied as a separate file. We leave it to the decision of the editors whether this should be part of the main manuscript as table 2, or a supplementary table.

Discussion:

Emphasized the importance of TVF and BVF in renal oxygenations parameters.

Line 232: Great way of highlighting the need of renal functions assessment in clinical settings.

o Consider adding how this technique could also be utilize for other renal diseases progress-sions and risk stratifications, maybe the use expands beyond that for renal transplantation.

>>>>> We have added the following statement in the Discussion section:

"We envision that stand-alone MR oximetry will become a clinical tool for diagnosis and risk stratification of AKI resulting from other causes aside from ischemia/reperfusion. It may also prove beneficial for early diagnosis and for monitoring disease progression in several other renal disorders."

Line 297: 9.4T -> clinical scanner (1.5 or 3T): consider adding citations for similar studies, both in-vivo and preclinical, on renal oxygenation done before.

>>> For a comprehensive overview of similar work we have added a reference to a review published last year in Nature Reviews Nephrology.

o Consider expanding on highlighting the difficulty of clinical translations and novelty of this study in direct renal oxygenations comparisons.

>>> We thank the reviewer for stressing the importance of clinical translation. The platform itself will be of limited use in a clinical setting because of the invasive nature of the reference methods. However, we are continuously working to transfer the results and gained understanding of renal physiology and MR methodology to apply in clinical settings, for the ultimate goal of benefiting patients.

Method:
Absorption coefficient remarks have been answered.
Remarks on coupling uncertainties was explained in rebuttal letter and manuscript.

Line 437: What would cause the difference in baseline concentrations? Since these negative concentrations were only observed in some of the measurements.
o Could these negative concentrations be caused by using anaesthesia or probe introduction (methodological) or physiological variation in the mice model?
>>>>> The variability of baseline concentrations has many causes. Physiologically the kidney has a high degree of inhomogeneity, due to its complex vascularization. This leads to phenomena like shunt diffusions of oxygen from the arteries to the veins. A major source of instrumental variability is uncertainties in the light coupling from the fibers to the tissue, and vice versa. In our continuous wave setup these uncertainties lead to imperfect discrimination between absorption and scattering, which affects the absolute concentration estimates. Underestimates of baseline concentrations together with more precisely measured strong concentration changes can lead to negative concentration estimates in a first iterative step. In an additional step, this problem was resolved by correcting the baseline concentrations using the more precise information about the concentration changes.

Line 509: clarified the use of maximal distance between NIRS and PHYSIOL probes.
The choice of occlusion time (230 sec) was clarified methodologically.
>>>>> We thank the reviewer for his comments.
