# Supplementary material for: MRI-PHYSIOL-NIRS: an integrated platform to study kidney oxygenation
Source: Commun Eng. 2026 Oct 16;5:176. doi: 10.1038/s44172-026-00799-x (PMC13675240; doi:10.1038/s44172-026-00799-x)
Supplement: Supplementary file 2 [file 44172_2026_799_MOESM2_ESM.pdf]

Supplemental Table 1: repeated-measures correlations

	**baseline**					
	renal cortex					
	parameter 1	parameter 2	rmcorr r	R^2	nominal p-value	adjusted p-value
1	T_2	T_2*	**-0,379**	**0,144**	**6,10E-03**	**3,00E-02**
2	T_2	deoxyHb	0,132	0,017	3,67E-01	5,46E-01
3	T_2	TVF	**-0,549**	**0,302**	**2,98E-05**	**2,34E-04**
4	T_2	kidney size	-0,194	0,038	1,72E-01	3,38E-01
5	T_2	BVF	0,035	0,001	8,19E-01	9,01E-01
6	T_2	TotalHb	0,128	0,016	4,02E-01	5,57E-01
7	T_2	OxyHb	0,087	0,008	5,53E-01	7,07E-01
8	T_2	SO_2 MRI	-0,142	0,020	3,40E-01	5,20E-01
9	T_2	SO_2 NIRS	-0,239	0,057	1,06E-01	2,32E-01
10	T_2	pO_2	0,246	0,061	1,11E-01	2,32E-01
11	T_2*	deoxyHb	0,021	0,000	8,89E-01	9,40E-01
12	T_2*	TVF	0,309	0,096	2,73E-02	1,02E-01
13	T_2*	kidney size	-0,157	0,025	2,72E-01	4,68E-01
14	T_2*	BVF	0,350	0,123	1,84E-02	7,80E-02
15	T_2*	TotalHb	**-0,445**	**0,198**	**2,22E-03**	**1,40E-02**
16	T_2*	OxyHb	**-0,425**	**0,180**	**2,35E-03**	**1,40E-02**
17	T_2*	SO_2 MRI	**0,576**	**0,331**	**2,31E-05**	**2,12E-04**
18	T_2*	SO_2 NIRS	-0,182	0,033	2,20E-01	4,03E-01
19	T_2*	pO_2	0,195	0,038	2,11E-01	4,00E-01
20	deoxyHb	TVF	0,004	0,000	9,78E-01	9,78E-01
21	deoxyHb	kidney size	-0,122	0,015	4,05E-01	5,57E-01
22	deoxyHb	BVF	0,011	0,000	9,45E-01	9,68E-01
23	deoxyHb	TotalHb	-0,027	0,001	8,55E-01	9,22E-01
24	deoxyHb	OxyHb	-0,248	0,062	7,92E-02	2,03E-01
25	deoxyHb	SO_2 MRI	-0,060	0,004	6,96E-01	8,12E-01
26	deoxyHb	SO_2 NIRS	**-0,648**	**0,420**	**4,75E-07**	**6,53E-06**
27	deoxyHb	pO_2	-0,010	0,000	9,51E-01	9,68E-01
28	TVF	kidney size	0,054	0,003	7,09E-01	8,12E-01
29	TVF	BVF	**0,423**	**0,179**	**3,77E-03**	**2,10E-02**
30	TVF	TotalHb	-0,282	0,080	6,04E-02	1,81E-01
31	TVF	OxyHb	-0,268	0,072	6,24E-02	1,81E-01
32	TVF	SO_2 MRI	**0,389**	**0,151**	**6,91E-03**	**3,20E-02**
33	TVF	SO_2 NIRS	-0,234	0,055	1,14E-01	2,32E-01
34	TVF	pO_2	-0,161	0,026	3,01E-01	4,74E-01
35	kidney size	BVF	-0,161	0,026	2,90E-01	4,74E-01
36	kidney size	TotalHb	0,075	0,006	6,27E-01	7,49E-01
37	kidney size	OxyHb	0,110	0,012	4,53E-01	6,07E-01
38	kidney size	SO_2 MRI	-0,256	0,066	8,23E-02	2,03E-01
39	kidney size	SO_2 NIRS	0,251	0,063	8,86E-02	2,03E-01
40	kidney size	pO_2	0,036	0,001	8,17E-01	9,01E-01
41	BVF	TotalHb	-0,283	0,080	8,13E-02	2,03E-01
42	BVF	OxyHb	-0,296	0,088	5,38E-02	1,74E-01
43	BVF	SO_2 MRI	**0,771**	**0,595**	**5,76E-10**	**1,06E-08**

44	BVF	SO_2 NIRS	-0,272	0,074	8,54E-02	2,03E-01
45	BVF	pO_2	0,093	0,009	5,85E-01	7,22E-01
46	TotalHb	**OxyHb**	**0,967**	**0,935**	**2,45E-28**	**1,35E-26**
47	TotalHb	SO_2 MRI	-0,344	0,118	2,79E-02	1,02E-01
48	TotalHb	**SO_2 NIRS**	**0,647**	**0,419**	**1,56E-06**	**1,71E-05**
49	TotalHb	pO_2	-0,202	0,041	2,31E-01	4,10E-01
50	OxyHb	SO_2 MRI	-0,319	0,102	3,29E-02	1,13E-01
51	OxyHb	**SO_2 NIRS**	**0,794**	**0,631**	**9,66E-12**	**2,66E-10**
52	OxyHb	pO_2	-0,167	0,028	2,96E-01	4,74E-01
53	SO_2 MRI	SO_2 NIRS	-0,136	0,019	3,84E-01	5,55E-01
54	SO_2 MRI	pO_2	0,099	0,010	5,49E-01	7,07E-01
55	SO_2 NIRS	pO_2	-0,086	0,007	5,91E-01	7,22E-01

baseline

renal medulla

	parameter 1	parameter 2	rmcorr r	R^2	nominal p-value	adjusted p-value
1	T_2	T_2*	0,117	0,014	4,12E-01	7,55E-01
2	T_2	deoxyHb	-0,224	0,050	1,30E-01	2,99E-01
3	T_2	TVF	-0,356	0,127	1,03E-02	5,13E-02
4	T_2	kidney size	-0,288	0,083	4,02E-02	1,24E-01
5	T_2	BVF	-0,131	0,017	3,91E-01	7,41E-01
6	T_2	TotalHb	-0,066	0,004	6,53E-01	9,06E-01
7	T_2	OxyHb	0,001	0,000	9,96E-01	9,96E-01
8	T_2	SO_2 MRI	0,033	0,001	8,27E-01	9,48E-01
9	T_2	SO_2 NIRS	0,098	0,010	5,02E-01	7,90E-01
10	T_2	pO_2	0,030	0,001	8,58E-01	9,60E-01
11	T_2*	deoxyHb	0,009	0,000	9,52E-01	9,87E-01
12	T_2*	TVF	0,204	0,042	1,51E-01	3,20E-01
13	T_2*	kidney size	-0,106	0,011	4,59E-01	7,68E-01
14	T_2*	BVF	**0,540**	**0,292**	**1,27E-04**	**1,17E-03**
15	T_2*	TotalHb	**0,461**	**0,212**	**8,68E-04**	**5,31E-03**
16	T_2*	OxyHb	**0,499**	**0,249**	**2,65E-04**	**1,83E-03**
17	T_2*	SO_2 MRI	**0,597**	**0,357**	**9,26E-06**	**1,27E-04**
18	T_2*	SO_2 NIRS	0,294	0,086	4,06E-02	1,24E-01
19	T_2*	pO_2	-0,006	0,000	9,69E-01	9,87E-01
20	deoxyHb	TVF	-0,017	0,000	9,07E-01	9,60E-01
21	deoxyHb	kidney size	-0,053	0,003	7,25E-01	9,06E-01
22	deoxyHb	BVF	-0,060	0,004	7,08E-01	9,06E-01
23	deoxyHb	TotalHb	**0,533**	**0,285**	**7,93E-05**	**8,72E-04**
24	deoxyHb	OxyHb	0,302	0,091	3,49E-02	1,20E-01
25	deoxyHb	SO_2 MRI	-0,064	0,004	6,82E-01	9,06E-01
26	deoxyHb	SO_2 NIRS	-0,216	0,047	1,36E-01	2,99E-01
27	deoxyHb	pO_2	0,066	0,004	7,00E-01	9,06E-01
28	TVF	kidney size	0,237	0,056	9,41E-02	2,35E-01

29	TVF	BVF	0,135	0,018	3,78E-01	7,41E-01
30	TVF	TotalHb	0,217	0,047	1,34E-01	2,99E-01
31	TVF	OxyHb	0,243	0,059	9,24E-02	2,35E-01
32	TVF	SO_2 MRI	0,180	0,032	2,26E-01	4,61E-01
33	TVF	SO_2 NIRS	**0,439**	**0,192**	**1,62E-03**	**8,90E-03**
34	TVF	pO_2	0,079	0,006	6,31E-01	9,06E-01
35	kidney size	BVF	0,018	0,000	9,07E-01	9,60E-01
36	kidney size	TotalHb	0,036	0,001	8,05E-01	9,48E-01
37	kidney size	OxyHb	0,065	0,004	6,58E-01	9,06E-01
38	kidney size	SO_2 MRI	0,020	0,000	8,93E-01	9,60E-01
39	kidney size	SO_2 NIRS	-0,033	0,001	8,23E-01	9,48E-01
40	kidney size	pO_2	-0,127	0,016	4,42E-01	7,68E-01
41	BVF	TotalHb	0,358	0,128	1,86E-02	7,85E-02
42	BVF	OxyHb	0,381	0,145	1,17E-02	5,38E-02
43	BVF	SO_2 MRI	**0,796**	**0,634**	**6,09E-11**	**1,67E-09**
44	BVF	SO_2 NIRS	0,324	0,105	3,41E-02	1,20E-01
45	BVF	pO_2	0,071	0,005	6,96E-01	9,06E-01
46	TotalHb	**OxyHb**	**0,961**	**0,923**	**5,84E-29**	**3,21E-27**
47	TotalHb	SO_2 MRI	0,302	0,091	4,38E-02	1,27E-01
48	TotalHb	**SO_2 NIRS**	**0,491**	**0,241**	**2,53E-04**	**1,83E-03**
49	TotalHb	pO_2	0,122	0,015	4,61E-01	7,68E-01
50	OxyHb	SO_2 MRI	0,338	0,114	2,33E-02	9,15E-02
51	OxyHb	**SO_2 NIRS**	**0,629**	**0,396**	**7,50E-07**	**1,37E-05**
52	OxyHb	pO_2	0,116	0,014	4,80E-01	7,77E-01
53	SO_2 MRI	SO_2 NIRS	0,287	0,082	5,60E-02	1,54E-01
54	SO_2 MRI	pO_2	0,077	0,006	6,62E-01	9,06E-01
55	SO_2 NIRS	pO_2	-0,046	0,002	7,82E-01	9,48E-01

	**aortic occlusion**					
	renal cortex					
	parameter 1	parameter 2	rmcorr r	R^2	nominal p-value	adjusted p-value
1	T_2	T_2*	**0,869**	**0,755**	**3,32E-29**	**1,82E-27**
2	T_2	deoxyHb	**-0,830**	**0,689**	**9,64E-23**	**1,06E-21**
3	T_2	TVF	**0,797**	**0,636**	**1,95E-21**	**1,79E-20**
4	T_2	kidney size	**0,826**	**0,683**	**3,56E-24**	**4,90E-23**
5	T_2	BVF	**0,609**	**0,371**	**6,21E-10**	**1,18E-09**
6	T_2	TotalHb	**0,251**	**0,063**	**2,68E-02**	**2,89E-02**
7	T_2	OxyHb	**0,574**	**0,329**	**9,38E-09**	**1,52E-08**
8	T_2	SO_2 MRI	**0,741**	**0,549**	**5,20E-16**	**2,20E-15**
9	T_2	SO_2 NIRS	**0,748**	**0,560**	**2,79E-16**	**1,28E-15**
10	T_2	pO_2	**0,753**	**0,567**	**7,04E-13**	**2,28E-12**
11	T_2*	deoxyHb	**-0,761**	**0,578**	**3,06E-17**	**1,68E-16**
12	T_2*	TVF	**0,622**	**0,387**	**3,68E-11**	**9,64E-11**

13	T_2*	kidney size	**0,718**	**0,516**	**7,82E-16**	**3,07E-15**
14	T_2*	BVF	**0,466**	**0,218**	**6,81E-06**	**8,71E-06**
15	T_2*	TotalHb	0,035	0,001	7,61E-01	7,61E-01
16	T_2*	OxyHb	**0,365**	**0,133**	**5,91E-04**	**6,77E-04**
17	T_2*	SO_2 MRI	**0,676**	**0,457**	**1,30E-12**	**3,98E-12**
18	T_2*	SO_2 NIRS	**0,546**	**0,298**	**7,93E-08**	**1,12E-07**
19	T_2*	pO_2	**0,635**	**0,403**	**1,78E-08**	**2,65E-08**
20	deoxyHb	TVF	**-0,663**	**0,440**	**4,58E-12**	**1,33E-11**
21	deoxyHb	kidney size	**-0,611**	**0,373**	**5,49E-10**	**1,08E-09**
22	deoxyHb	BVF	**-0,648**	**0,420**	**1,43E-10**	**3,01E-10**
23	deoxyHb	TotalHb	-0,186	0,035	1,03E-01	1,07E-01
24	deoxyHb	OxyHb	**-0,606**	**0,367**	**7,86E-10**	**1,40E-09**
25	deoxyHb	SO_2 MRI	**-0,788**	**0,622**	**1,05E-17**	**6,39E-17**
26	deoxyHb	SO_2 NIRS	**-0,791**	**0,626**	**3,24E-19**	**2,54E-18**
27	deoxyHb	pO_2	**-0,681**	**0,464**	**5,46E-09**	**9,11E-09**
28	TVF	kidney size	**0,690**	**0,476**	**2,73E-14**	**9,40E-14**
29	TVF	BVF	**0,530**	**0,281**	**1,77E-07**	**2,38E-07**
30	TVF	TotalHb	0,148	0,022	1,95E-01	1,98E-01
31	TVF	OxyHb	**0,437**	**0,191**	**2,86E-05**	**3,50E-05**
32	TVF	SO_2 MRI	**0,628**	**0,394**	**1,24E-10**	**2,73E-10**
33	TVF	SO_2 NIRS	**0,632**	**0,400**	**1,10E-10**	**2,52E-10**
34	TVF	pO_2	**0,519**	**0,270**	**1,09E-05**	**1,36E-05**
35	kidney size	BVF	**0,531**	**0,282**	**1,75E-07**	**2,38E-07**
36	kidney size	TotalHb	**0,378**	**0,143**	**6,54E-04**	**7,34E-04**
37	kidney size	OxyHb	**0,563**	**0,317**	**2,02E-08**	**2,92E-08**
38	kidney size	SO_2 MRI	**0,607**	**0,369**	**7,09E-10**	**1,30E-09**
39	kidney size	SO_2 NIRS	**0,633**	**0,401**	**1,01E-10**	**2,42E-10**
40	kidney size	pO_2	**0,720**	**0,518**	**2,08E-11**	**5,73E-11**
41	BVF	TotalHb	**0,459**	**0,210**	**5,76E-05**	**6,89E-05**
42	BVF	OxyHb	**0,661**	**0,437**	**4,50E-11**	**1,12E-10**
43	BVF	SO_2 MRI	**0,792**	**0,628**	**5,73E-18**	**3,94E-17**
44	BVF	SO_2 NIRS	**0,750**	**0,563**	**4,07E-15**	**1,49E-14**
45	BVF	pO_2	**0,447**	**0,200**	**4,86E-04**	**5,68E-04**
46	TotalHb	**OxyHb**	**0,878**	**0,770**	**5,72E-26**	**1,05E-24**
47	TotalHb	SO_2 MRI	0,219	0,048	6,48E-02	6,85E-02
48	TotalHb	**SO_2 NIRS**	**0,644**	**0,415**	**2,56E-10**	**5,22E-10**
49	TotalHb	pO_2	**0,378**	**0,143**	**6,86E-03**	**7,55E-03**
50	OxyHb	SO_2 MRI	**0,589**	**0,347**	**1,38E-08**	**2,10E-08**
51	OxyHb	**SO_2 NIRS**	**0,874**	**0,764**	**1,95E-27**	**5,36E-26**
52	OxyHb	pO_2	**0,625**	**0,391**	**1,98E-07**	**2,59E-07**
53	SO_2 MRI	SO_2 NIRS	**0,774**	**0,599**	**9,81E-17**	**4,90E-16**
54	SO_2 MRI	pO_2	**0,653**	**0,426**	**1,21E-08**	**1,89E-08**
55	SO_2 NIRS	pO_2	**0,683**	**0,466**	**4,85E-09**	**8,34E-09**

Supplemental Table 1: repeated-measures correlations

	**aortic occlusion**					
	renal medulla					
	parameter 1	parameter 2	rmcorr r	R^2	nominal p-value	adjusted p-value
1	T_2	T_2*	**0,914**	**0,835**	**5,79E-37**	**1,59E-35**
2	T_2	deoxyHb	-0,158	0,025	1,67E-01	1,80E-01
3	T_2	TVF	**0,843**	**0,711**	**5,71E-26**	**7,86E-25**
4	T_2	kidney size	**0,795**	**0,633**	**2,80E-21**	**1,92E-20**
5	T_2	BVF	**0,561**	**0,315**	**2,37E-08**	**4,08E-08**
6	T_2	TotalHb	**0,679**	**0,461**	**8,09E-12**	**2,12E-11**
7	T_2	OxyHb	**0,740**	**0,548**	**9,64E-15**	**3,31E-14**
8	T_2	SO_2 MRI	**0,752**	**0,565**	**5,93E-18**	**2,97E-17**
9	T_2	SO_2 NIRS	**0,759**	**0,576**	**8,37E-16**	**3,29E-15**
10	T_2	pO_2	**0,694**	**0,482**	**1,97E-10**	**4,18E-10**
11	T_2*	deoxyHb	**-0,239**	**0,057**	**3,54E-02**	**4,06E-02**
12	T_2*	TVF	**0,699**	**0,489**	**8,77E-15**	**3,21E-14**
13	T_2*	kidney size	**0,728**	**0,529**	**2,13E-16**	**8,99E-16**
14	T_2*	BVF	**0,596**	**0,355**	**1,80E-09**	**3,67E-09**
15	T_2*	TotalHb	**0,727**	**0,528**	**4,92E-14**	**1,50E-13**
16	T_2*	OxyHb	**0,810**	**0,657**	**2,52E-19**	**1,38E-18**
17	T_2*	SO_2 MRI	**0,818**	**0,669**	**2,53E-23**	**2,78E-22**
18	T_2*	SO_2 NIRS	**0,844**	**0,713**	**2,73E-22**	**2,14E-21**
19	T_2*	pO_2	**0,754**	**0,569**	**6,32E-13**	**1,83E-12**
20	deoxyHb	TVF	0,014	0,000	9,00E-01	9,00E-01
21	deoxyHb	kidney size	-0,181	0,033	1,14E-01	1,25E-01
22	deoxyHb	BVF	-0,160	0,026	1,82E-01	1,89E-01
23	deoxyHb	TotalHb	0,145	0,021	2,27E-01	2,32E-01
24	deoxyHb	OxyHb	-0,161	0,026	1,81E-01	1,89E-01
25	deoxyHb	SO_2 MRI	-0,224	0,050	4,82E-02	5,41E-02
26	deoxyHb	SO_2 NIRS	**-0,341**	**0,116**	**3,59E-03**	**4,38E-03**
27	deoxyHb	pO_2	**-0,310**	**0,096**	**2,87E-02**	**3,36E-02**
28	TVF	kidney size	**0,642**	**0,412**	**5,42E-12**	**1,49E-11**
29	TVF	BVF	**0,279**	**0,078**	**9,68E-03**	**1,16E-02**
30	TVF	TotalHb	**0,512**	**0,262**	**1,68E-06**	**2,44E-06**
31	TVF	OxyHb	**0,533**	**0,284**	**5,01E-07**	**7,88E-07**
32	TVF	SO_2 MRI	**0,487**	**0,238**	**8,35E-07**	**1,24E-06**
33	TVF	SO_2 NIRS	**0,509**	**0,259**	**1,97E-06**	**2,78E-06**
34	TVF	pO_2	**0,492**	**0,242**	**3,68E-05**	**4,70E-05**
35	kidney size	BVF	**0,462**	**0,213**	**8,69E-06**	**1,14E-05**
36	kidney size	TotalHb	**0,547**	**0,299**	**2,24E-07**	**3,63E-07**
37	kidney size	OxyHb	**0,602**	**0,363**	**5,37E-09**	**9,85E-09**
38	kidney size	SO_2 MRI	**0,619**	**0,383**	**4,81E-11**	**1,15E-10**
39	kidney size	SO_2 NIRS	**0,656**	**0,430**	**7,32E-11**	**1,68E-10**
40	kidney size	pO_2	**0,578**	**0,334**	**5,64E-07**	**8,62E-07**
41	BVF	TotalHb	**0,512**	**0,262**	**5,00E-06**	**6,77E-06**
42	BVF	OxyHb	**0,580**	**0,336**	**1,17E-07**	**1,95E-07**

43	BVF	SO_2 MRI	**0,825**	**0,681**	**2,71E-22**	**2,14E-21**
44	BVF	SO_2 NIRS	**0,640**	**0,409**	**1,88E-09**	**3,69E-09**
45	BVF	pO_2	**0,487**	**0,237**	**1,24E-04**	**1,56E-04**
46	TotalHb	**OxyHb**	**0,958**	**0,918**	**4,75E-43**	**2,61E-41**
47	TotalHb	SO_2 MRI	**0,649**	**0,421**	**1,32E-10**	**2,90E-10**
48	TotalHb	**SO_2 NIRS**	**0,833**	**0,694**	**3,18E-21**	**1,94E-20**
49	TotalHb	pO_2	**0,563**	**0,317**	**5,05E-06**	**6,77E-06**
50	OxyHb	SO_2 MRI	**0,735**	**0,540**	**1,83E-14**	**5,93E-14**
51	OxyHb	**SO_2 NIRS**	**0,936**	**0,876**	**3,54E-36**	**6,49E-35**
52	OxyHb	pO_2	**0,680**	**0,463**	**5,92E-09**	**1,05E-08**
53	SO_2 MRI	SO_2 NIRS	**0,785**	**0,616**	**1,81E-17**	**8,30E-17**
54	SO_2 MRI	pO_2	**0,713**	**0,508**	**4,02E-11**	**1,01E-10**
55	SO_2 NIRS	pO_2	**0,689**	**0,475**	**3,12E-09**	**5,93E-09**

hyperoxia

renal cortex

	parameter 1	parameter 2	rmcorr r	R^2	nominal p-value	adjusted p-value
1	T_2	T_2*	**0,720**	**0,518**	**3,14E-12**	**4,32E-11**
2	T_2	deoxyHb	**-0,582**	**0,339**	**1,54E-07**	**1,06E-06**
3	T_2	TVF	-0,181	0,033	1,37E-01	2,42E-01
4	T_2	kidney size	-0,006	0,000	9,63E-01	9,63E-01
5	T_2	BVF	**-0,509**	**0,259**	**5,29E-05**	**2,24E-04**
6	T_2	TotalHb	**-0,547**	**0,299**	**1,17E-06**	**7,12E-06**
7	T_2	OxyHb	0,102	0,010	4,06E-01	5,31E-01
8	T_2	SO_2 MRI	-0,085	0,007	5,29E-01	6,36E-01
9	T_2	SO_2 NIRS	**0,324**	**0,105**	**6,67E-03**	**1,80E-02**
10	T_2	pO_2	**0,469**	**0,220**	**7,71E-04**	**2,00E-03**
11	T_2*	deoxyHb	**-0,713**	**0,508**	**6,39E-12**	**7,03E-11**
12	T_2*	TVF	-0,237	0,056	4,98E-02	1,02E-01
13	T_2*	kidney size	0,059	0,003	6,32E-01	7,24E-01
14	T_2*	BVF	**-0,341**	**0,116**	**9,53E-03**	**2,50E-02**
15	T_2*	TotalHb	**-0,531**	**0,282**	**2,66E-06**	**1,33E-05**
16	T_2*	OxyHb	0,240	0,057	4,73E-02	1,00E-01
17	T_2*	SO_2 MRI	0,171	0,029	2,03E-01	3,02E-01
18	T_2*	SO_2 NIRS	**0,482**	**0,232**	**2,76E-05**	**1,27E-04**
19	T_2*	pO_2	**0,716**	**0,512**	**1,09E-08**	**8,58E-08**
20	deoxyHb	TVF	0,087	0,008	4,76E-01	6,03E-01
21	deoxyHb	kidney size	-0,254	0,065	3,51E-02	7,70E-02
22	deoxyHb	BVF	**0,445**	**0,198**	**5,26E-04**	**2,00E-03**
23	deoxyHb	TotalHb	**0,407**	**0,165**	**2,19E-04**	**8,04E-04**
24	deoxyHb	OxyHb	**-0,667**	**0,445**	**2,65E-11**	**2,43E-10**
25	deoxyHb	SO_2 MRI	0,049	0,002	7,18E-01	8,06E-01
26	deoxyHb	SO_2 NIRS	**-0,862**	**0,743**	**3,77E-24**	**1,04E-22**
27	deoxyHb	pO_2	**-0,618**	**0,383**	**1,70E-06**	**9,33E-06**

28	TVF	kidney size	0,177	0,031	1,45E-01	2,49E-01
29	TVF	BVF	0,153	0,023	2,56E-01	3,61E-01
30	TVF	TotalHb	-0,132	0,018	2,78E-01	3,83E-01
31	TVF	OxyHb	-0,194	0,037	1,11E-01	2,10E-01
32	TVF	SO_2 MRI	0,182	0,033	1,76E-01	2,76E-01
33	TVF	SO_2 NIRS	-0,226	0,051	6,22E-02	1,22E-01
34	TVF	pO_2	-0,187	0,035	2,02E-01	3,02E-01
35	kidney size	BVF	-0,183	0,033	1,73E-01	2,76E-01
36	kidney size	TotalHb	-0,112	0,012	3,61E-01	4,85E-01
37	kidney size	OxyHb	0,150	0,023	2,18E-01	3,15E-01
38	kidney size	SO_2 MRI	-0,095	0,009	4,83E-01	6,03E-01
39	kidney size	SO_2 NIRS	0,184	0,034	1,30E-01	2,38E-01
40	kidney size	pO_2	0,038	0,001	7,99E-01	8,62E-01
41	BVF	TotalHb	0,304	0,092	2,16E-02	5,20E-02
42	BVF	OxyHb	-0,184	0,034	1,71E-01	2,76E-01
43	BVF	SO_2 MRI	**0,774**	**0,599**	**1,68E-12**	**3,08E-11**
44	BVF	SO_2 NIRS	-0,287	0,082	3,06E-02	7,00E-02
45	BVF	pO_2	-0,384	0,147	2,09E-02	5,20E-02
46	TotalHb	**OxyHb**	**0,410**	**0,168**	**1,97E-04**	**7,72E-04**
47	TotalHb	SO_2 MRI	-0,027	0,001	8,41E-01	8,73E-01
48	TotalHb	**SO_2 NIRS**	0,024	0,001	8,36E-01	8,73E-01
49	TotalHb	pO_2	0,020	0,000	8,89E-01	9,05E-01
50	OxyHb	SO_2 MRI	-0,071	0,005	5,98E-01	7,00E-01
51	OxyHb	**SO_2 NIRS**	**0,880**	**0,775**	**2,45E-26**	**1,35E-24**
52	OxyHb	pO_2	**0,444**	**0,197**	**1,24E-03**	**4,00E-03**
53	SO_2 MRI	SO_2 NIRS	-0,043	0,002	7,53E-01	8,29E-01
54	SO_2 MRI	pO_2	-0,108	0,012	5,32E-01	6,36E-01
55	SO_2 NIRS	pO_2	**0,491**	**0,241**	**2,99E-04**	**1,00E-03**

hyperoxia

renal medulla

	parameter 1	parameter 2	rmcorr r	R^2	nominal p-value	adjusted p-value
1	T_2	T_2*	**0,823**	**0,678**	**3,92E-18**	**7,19E-17**
2	T_2	deoxyHb	**-0,635**	**0,403**	**4,61E-09**	**2,54E-08**
3	T_2	TVF	**-0,318**	**0,101**	**7,79E-03**	**1,48E-02**
4	T_2	kidney size	0,051	0,003	6,79E-01	8,12E-01
5	T_2	BVF	**-0,591**	**0,349**	**1,32E-06**	**4,83E-06**
6	T_2	TotalHb	0,227	0,052	6,06E-02	9,81E-02
7	T_2	OxyHb	**0,566**	**0,320**	**4,09E-07**	**1,61E-06**
8	T_2	SO_2 MRI	0,046	0,002	7,35E-01	8,16E-01
9	T_2	SO_2 NIRS	**0,683**	**0,466**	**1,05E-10**	**7,19E-10**
10	T_2	pO_2	**0,746**	**0,556**	**2,18E-08**	**9,97E-08**
11	T_2*	deoxyHb	**-0,685**	**0,469**	**8,52E-11**	**6,69E-10**
12	T_2*	TVF	**-0,391**	**0,153**	**8,83E-04**	**2,02E-03**

Supplemental Table 1: repeated-measures correlations

13	T_2*	kidney size	0,129	0,017	2,91E-01	3,81E-01
14	T_2*	BVF	-0,231	0,053	8,38E-02	1,25E-01
15	T_2*	TotalHb	**0,364**	**0,133**	**2,11E-03**	**4,63E-03**
16	T_2*	OxyHb	**0,717**	**0,514**	**4,39E-12**	**4,03E-11**
17	T_2*	SO_2 MRI	**0,476**	**0,227**	**1,81E-04**	**4,74E-04**
18	T_2*	SO_2 NIRS	**0,770**	**0,592**	**1,09E-14**	**1,20E-13**
19	T_2*	pO_2	**0,758**	**0,575**	**9,35E-09**	**4,67E-08**
20	deoxyHb	TVF	**0,281**	**0,079**	**1,95E-02**	**3,57E-02**
21	deoxyHb	kidney size	0,030	0,001	8,10E-01	8,73E-01
22	deoxyHb	BVF	0,232	0,054	8,23E-02	1,25E-01
23	deoxyHb	TotalHb	0,161	0,026	1,60E-01	2,25E-01
24	deoxyHb	OxyHb	**-0,433**	**0,187**	**7,51E-05**	**2,29E-04**
25	deoxyHb	SO_2 MRI	-0,285	0,081	3,19E-02	5,48E-02
26	deoxyHb	SO_2 NIRS	**-0,745**	**0,555**	**5,07E-15**	**6,97E-14**
27	deoxyHb	pO_2	**-0,571**	**0,326**	**1,51E-05**	**5,20E-05**
28	TVF	kidney size	0,045	0,002	7,15E-01	8,16E-01
29	TVF	BVF	0,204	0,041	1,29E-01	1,86E-01
30	TVF	TotalHb	**-0,322**	**0,103**	**7,05E-03**	**1,44E-02**
31	TVF	OxyHb	**-0,448**	**0,201**	**1,14E-04**	**3,14E-04**
32	TVF	SO_2 MRI	-0,244	0,060	6,69E-02	1,05E-01
33	TVF	SO_2 NIRS	**-0,392**	**0,154**	**8,55E-04**	**2,02E-03**
34	TVF	pO_2	-0,324	0,105	3,89E-02	6,48E-02
35	kidney size	BVF	-0,152	0,023	2,60E-01	3,49E-01
36	kidney size	TotalHb	0,043	0,002	7,27E-01	8,16E-01
37	kidney size	OxyHb	0,021	0,000	8,62E-01	9,11E-01
38	kidney size	SO_2 MRI	-0,018	0,000	8,93E-01	9,27E-01
39	kidney size	SO_2 NIRS	-0,011	0,000	9,31E-01	9,31E-01
40	kidney size	pO_2	-0,109	0,012	4,96E-01	6,20E-01
41	BVF	TotalHb	0,045	0,002	7,42E-01	8,16E-01
42	BVF	OxyHb	-0,087	0,008	5,20E-01	6,35E-01
43	BVF	SO_2 MRI	**0,691**	**0,477**	**2,72E-09**	**1,66E-08**
44	BVF	SO_2 NIRS	-0,158	0,025	2,39E-01	3,29E-01
45	BVF	pO_2	**-0,706**	**0,498**	**1,90E-05**	**6,14E-05**
46	TotalHb	**OxyHb**	**0,820**	**0,673**	**4,17E-20**	**1,15E-18**
47	TotalHb	SO_2 MRI	0,291	0,085	2,82E-02	5,00E-02
48	TotalHb	**SO_2 NIRS**	**0,431**	**0,185**	**8,33E-05**	**2,41E-04**
49	TotalHb	pO_2	0,102	0,010	4,79E-01	6,13E-01
50	OxyHb	SO_2 MRI	**0,380**	**0,145**	**3,53E-03**	**7,47E-03**
51	OxyHb	**SO_2 NIRS**	**0,825**	**0,681**	**1,51E-20**	**8,28E-19**
52	OxyHb	pO_2	**0,494**	**0,244**	**2,71E-04**	**6,77E-04**
53	SO_2 MRI	SO_2 NIRS	**0,349**	**0,122**	**7,73E-03**	**1,48E-02**
54	SO_2 MRI	pO_2	-0,021	0,000	9,15E-01	9,31E-01
55	SO_2 NIRS	pO_2	**0,674**	**0,455**	**7,87E-08**	**3,33E-07**

Supplemental Table 2: p-values obtained from non-parametric repeated-measures Friedman test followed by Dunn's post-hoc test for the statistical significance of the changes happening during the interventions shown in Fig.4a,b, Fig.5a,b

Parameter	aortic occlusion[a]		hyperoxia[a]	
	cortex	medulla	cortex	medulla
T2	1.80e-04	1.80e-04	0.016	0.015
T2*	3.80e-04	2.28e-04	0.033	0.018
deoxyHb	6.21e-04	0.940	0.009	0.016
TVF	2.85e-04	2.28e-04	0.752	0.063
BVF	5.69e-04	5.69e-04	0.029	0.133
totalHb	0.023	0.002	0.588	0.511
oxyHb	5.26e-04	0.002	0.294	0.065
StO_2MRI	4.27e-04	4.88e-04	0.945	0.487
StO_2NIRS	5.26e-04	0.003	0.027	0.004
pO_2	0.003	0.003	0.003	0.03
kidney size[b]	0.009		0.980	

[a] p-values obtained from non-parametric repeated-measures Friedman test, Dunn's post-hoc test

[b] kidney size was measured over the entire kidney, not separately for cortex and medulla
